# Adaptive resistance in cancer immunotherapy

**DOI:** 10.1038/s41423-026-01424-9

**Published:** 2026-05-20

**Authors:** Ke Yang, Chunqian Yang, Kai Xiong, Jiangtao Hao, Lilin Ye

**Affiliations:** 1 Institute of Immunological Innovation and Translation, Chongqing Medical University, Chongqing, China; 2 Department of Urology, The First Affiliated Hospital of Chongqing Medical University, Chongqing, China; 3 Institute of Immunology, Third Military Medical University, Chongqing, China; 4 Changping Laboratory, Beijing, China

**Keywords:** Adaptive resistance, Cancer immunotherapy, Antitumor immunological memory, T-cell exhaustion, Cancer therapeutic resistance, Immunological memory, Cancer immunotherapy

## Abstract

Despite revolutionizing oncology, cancer immunotherapy benefits only a minority of patients partly because of adaptive resistance. This resistance tends to manifest in two distinct clinical scenarios. The first is characterized by disease progression during treatment following an initial response, driven primarily by insufficient tumor-killing capacity. The second is characterized by relapse after initial remission, resulting from a failure to establish durable antitumor immune memory (AIM). Underlying both patterns is the progressive functional exhaustion of tumor-specific T cells, which reflects a dynamic equilibrium between immune attack and tumor evasion, a balance that is continuously challenged within an immunosuppressive tumor microenvironment (TME) sculpted by immunoediting. This review provides a comprehensive synthesis of current knowledge on adaptive resistance to cancer immunotherapy, with a particular emphasis on the mechanistic contributions of dysfunctional T-cell responses. We begin by outlining the frameworks of cancer immunoediting and AIM, detailing how exhausted T cells undermine sustained immunotherapeutic efficacy, and summarizing the achievements and limitations of current regimens. We then delineate how T-cell dysfunction, particularly within the TME and tumor-draining lymph nodes, culminates in adaptive resistance to immunotherapy, manifesting as either insufficient tumor-killing capacity leading to on-treatment progression or failure to establish durable AIM resulting in postremission relapse. A systematic analysis of the cellular and molecular drivers of this exhausted state follows, integrating established paradigms and highlighting key knowledge gaps. Finally, we critically evaluate emerging rejuvenation strategies, translational approaches for next-generation therapies, persistent challenges and promising directions for future research.

## Introduction

**Fig. 1:**
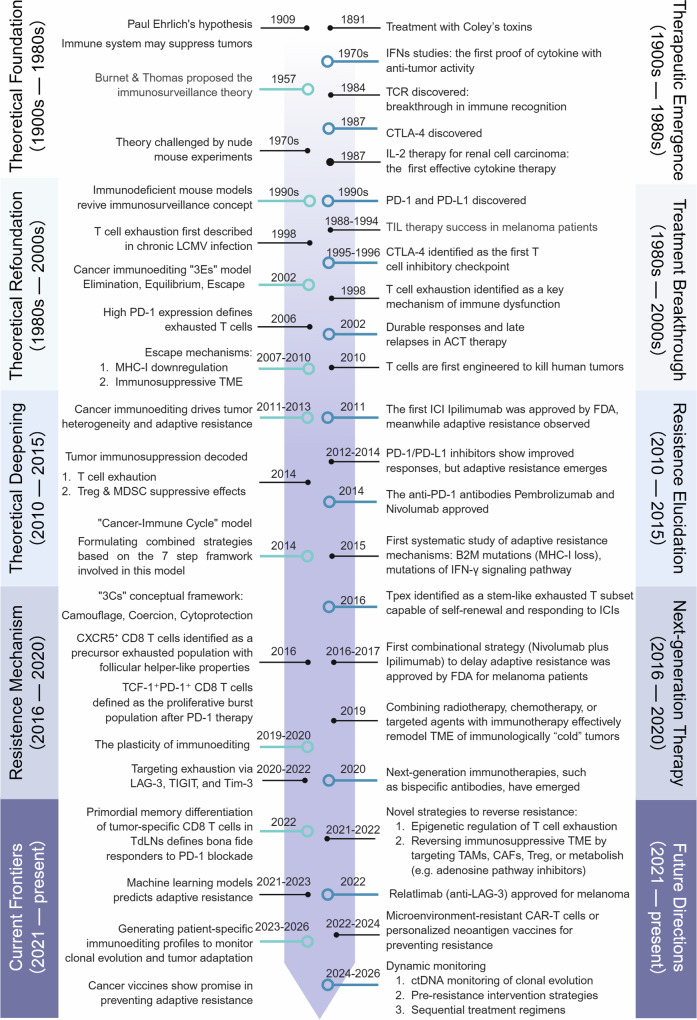
Milestones and roadblocks in cancer immunotherapy. This timeline illustrates the parallel evolution of cancer immunotherapy from initial proof-of-concept studies (left axis) to transformative clinical applications (right axis). A central theme is that each major therapeutic advance exerts selective pressure on tumors, driving the emergence of adaptive resistance mechanisms and, in turn, catalyzing the development of next-generation, more precise immune treatment strategies. Thus, the progression of immunotherapy reflects a continuous, dynamic interplay between therapeutic intervention and tumor evolutionary adaptation

Recent advances in tumor immune biology have driven a paradigm shift in oncology, moving therapeutic focus from directly targeting the tumor itself to harnessing the host immune system [1]. Discoveries in immune checkpoint regulation and breakthroughs in immune cell engineering have given rise to the next generation of cancer immunotherapies, notably immune checkpoint inhibitory (ICI) and immune cell therapy (ICT) [2–7]. ICI therapy, particularly targeting programmed cell death receptor 1 (PD-1) and its ligand B7-H1 (B7 homolog 1, also known as PD-L1), has emerged as a cornerstone of this revolution by effectively unleashing T-cell-mediated antitumor responses and yielding durable clinical efficacy in a subset of patients [8]. The therapeutic promise of immunotherapy relies on the conceptual framework of cancer immunoediting, which describes how the immune system dynamically shapes tumors through the elimination, equilibrium, and escape phases [9–12]. Within this framework, antitumor immune memory (AIM) represents a desired outcome of the elimination phase, contributing to long-term tumor control and patient survival. However, the induction and maintenance of AIM are inherently constrained, as tumors frequently progress to the escape phase through evolution of adaptive resistance mechanisms. Clinically, this resistance manifests in two distinct patterns, arising from both the progressive functional exhaustion of tumor-specific T cells and the dynamic equilibrium between immune attack and tumor evasion, which are continuously challenged within an immunosuppressive tumor microenvironment (TME) sculpted by immunoediting. One is characterized by progression during treatment following an initial response, driven primarily by insufficient tumor-killing capacity; the other is characterized by relapse after initial remission, resulting from a failure to establish durable AIM. Mechanistically, these clinical patterns reflect the progressive dysfunction of tumor-reactive T cells within the TME and tumor-draining lymph nodes (TdLNs) [13, 14]. This dysfunction represents not a passive loss of immunity but an active immunosuppressive state that enables tumor escape under immunotherapeutic pressure, which can be conceptualized by a “three Cs” framework encompassing camouflage, coercion, and cytoprotection [15, 16]. Collectively, by acting as a dynamic selective pressure that shapes tumor immunogenicity and drives the evolution of resistance, immunoediting positions adaptive resistance as the central challenge of contemporary cancer immunotherapy. This interplay between foundational discovery and clinical challenges is illustrated in Fig. 1, which maps the parallel evolution of immunological principles and translational advances.

If immunotherapy aims to establish protective immune memory comparable to that induced by acute infection, then its failure to do so—coupled with the insufficient tumor-killing capacity that permits on-treatment progression—reveals fundamental limitations in current strategies and underscores the need for a mechanistic explanation. We propose that adaptive resistance to cancer immunotherapy arises from the progressive dysfunction of tumor-specific T cells, which manifests as two interrelated yet distinct defects. The first is a loss of immediate effector function that causes primary treatment failure; the second is a failure to establish durable antitumor immune memory that leads to postremission relapse. This dual-defect framework unifies heterogeneous clinical outcomes across patients and tumor types and reframes the therapeutic challenge. In this review, we evaluate evidence positioning T-cell dysfunction as a central determinant of therapeutic durability, dissect the mechanisms driving both effector exhaustion and memory failure, and discuss how this integrated understanding is informing next-generation combinatorial immunotherapies.

## Status quo of cancer immunotherapy

### ICI therapy, from mechanistic foundations to clinical realities

A key feature of immunogenic tumor rejection by ICIs is the induced durable memory response, which therefore depends on a full-bodied T-cell response to develop long-lasting antitumor immunity essential for durable cancer control [17]. This response depends on costimulatory signals and is tightly regulated by inhibitory immune checkpoints such as cytotoxic T-lymphocyte antigen-4 (CTLA-4) to prevent autoimmunity [18, 19]. The groundbreaking discovery that CTLA-4 blockade enables durable clinical responses, exemplified by the induction of a survival plateau by ipilimumab for a decade in approximately 21% of patients with advanced melanoma, laid the foundation for the use of ICIs as a therapeutic paradigm [2, 20–22]. However, CTLA-4 blockade potently enhances early immune priming at the cost of significant immune-related adverse events (irAEs), reflecting its systemic role in maintaining self-tolerance [23]. In parallel, the PD-1/PD-L1 pathway was identified as a pivotal “molecular brake” that acts selectively on preactivated T cells [24–26]. Tumors frequently upregulate PD-L1 to hijack this pathway, delivering a potent inhibitory signal that suppresses T-cell-mediated antitumor activity and promotes exhaustion [27–29]. This exhausted state is characterized by a hierarchical loss of cytokine production, cytolytic capacity, and proliferative potential. Critically, it impairs the generation of functional, long-lived memory T cells [30, 31]. Consequently, PD-1/PD-L1 blockade can reinvigorate exhausted T cells and has demonstrated durable clinical efficacy across multiple malignancies, including historically immunotherapy-refractory cancers [32–34]. Importantly, unlike CTLA-4 blockade, PD-1/PD-L1 blockade generally results in a more favorable safety profile, which is likely attributable to its more restricted activity [35, 36]. These advances have led to rapid succession to the approval of the anti-PD-1 antibodies pembrolizumab and nivolumab (both in 2014), followed by the anti-PD-L1 antibodies atezolizumab (in 2016) and durvalumab (in 2017), as second-line therapies for advanced melanoma and metastatic colorectal cancer (mCRC) [37].

The clinical impact of ICIs is profound in solid tumors, particularly melanoma, which serves as a paradigm for immunotherapy success [38, 39]. Combination therapy with nivolumab and ipilimumab has set a new efficacy benchmark, with long-term follow-up data showing a median overall survival (OS) of 71.9 months and establishing this regimen as the gold standard [36, 40–43]. These durable responses that reach a survival plateau serve as strong clinical evidence for the development of effective AIM. Beyond melanoma, ICI therapy has demonstrated encouraging antitumor efficacy in numerous cancers, and its application is expanding into neoadjuvant and adjuvant settings for early-stage diseases, such as triple-negative breast cancer (TNBC) and renal cell carcinoma (RCC) [44–54]. This shift toward treating earlier-stage disease represents a strategic emphasis on eradicating minimal residual disease and fostering durable immunological memory before the establishment of extensive, tumor-driven immunosuppressive networks.

### Neoadjuvant ICI therapy: a paradigm shift toward early intervention

The integration of ICIs into neoadjuvant therapy has transformed the management of early-stage solid tumors. This approach aims to eliminate minimal residual disease while activating systemic antitumor immunity before surgery. Critically, by inducing early clonal expansion and differentiation of antigen-specific T cells prior to the establishment of robust immunosuppressive networks, it promotes functional immune memory and establishes durable immune surveillance that underpins long-term disease-free survival following tumor resection. Pivotal support for this strategy comes from the phase III KEYNOTE-522 trial in early-stage TNBC, in which neoadjuvant pembrolizumab combined with platinum-based chemotherapy significantly increased pathological complete response (pCR) rates and event-free survival compared with chemotherapy alone [55]. This represents a milestone in immuno-oncology, successfully leading to standard clinical guideline updates, which established immunotherapy as a component of curative-intent treatment for early-stage disease.

Neoadjuvant immunotherapy is based on essential concepts in cancer immunoediting. Early intervention provides favorable conditions for inducing diverse and sustained tumor-specific T-cell responses when tumor antigenicity is still relatively preserved and before the TME develops strong immunosuppressive traits. Notably, it enhances both immediate antitumor immunity and prolonged antigen exposure, which supports the formation and maintenance of functional tumor-specific memory T cells. This ultimately fosters durable immunological memory that ensures continuous immune surveillance after surgery and reduces the risk of late recurrence. Moreover, neoadjuvant therapy-triggered tumor cell death in situ can act as an endogenous vaccination strategy, amplifying systemic immune surveillance and promoting the generation of durable antitumor immunological memory. Accordingly, clinical investigations of neoadjuvant immunotherapy are accelerating across diverse solid tumors, including TNBC, non-small cell lung cancer (NSCLC), RCC, and melanoma, encompassing both ICI monotherapy and combination regimens [56–58]. A notable advancement is the adoption of pathological response metrics, including major pathological response and pCR, as early surrogate endpoints predictive of long-term survival, thereby bridging immediate immunologic activity with sustained clinical outcomes [59, 60].

### ICT: engineered immunity and solid tumor challenge

In addition to ICIs, ICT represents another pillar of cancer immunotherapy. Pioneered with tumor-infiltrating lymphocytes (TILs), ICT has evolved with advances in T-cell engineering [61–64]. The central tenet of ICT involves the generation or expansion of tumor-specific T-cell populations that mediate tumor eradication and the establishment of durable protective memory [65]. Mechanistically, T-cell receptor (TCR)-based therapies recognize intracellular antigen peptides presented by major histocompatibility complex (MHC) molecules, whereas chimeric antigen receptor (CAR) T cells enable direct, MHC-unrestricted recognition of tumor surface antigens via a single-chain variable fragment [66]. The clinical breakthrough of CD19-directed CAR-T cells in B-cell acute lymphoblastic leukemia, which achieved complete remission rates of up to 90%, marked the dawn of a new era in cell-based immunotherapy and provided a definitive proof of concept for engineered T cells [67]. This milestone highlights the potential of eliciting a potent, de novo antitumor T-cell response. The field is now evolving through novel strategies, including bispecific CAR-T cells targeting CD19 and CD22, as well as CD38-specific CAR-T cells, which aim to increase therapeutic efficacy by countering mechanisms such as antigen escape [68, 69].

Nevertheless, translating these successes from hematologic to solid tumors faces foundational hurdles, such as an immunosuppressive TME, inefficient T-cell function and trafficking, and a limited repertoire of tumor-specific antigens [70, 71]. These impediments compromise not only initial cytolytic activity but also the long-term engraftment and functional persistence of memory T-cell populations, which are essential for long-term control. This concept is directly supported by preclinical evidence from ICT models. Studies have demonstrated that the adoptive transfer of memory-phenotype T cells, as opposed to effector T cells, can elicit more robust and persistent systemic antitumor CD8^+^ T-cell responses, resulting in superior long-term tumor control [72]. These findings underscore that the differentiation state of infused T cells critically governs therapeutic durability, highlighting the induction or maintenance of memory-like properties as an essential objective for next-generation ICT. In response, innovative approaches, such as engineering inhibitory-signal-resistant CAR-T cells or developing in vivo CAR-T-cell platforms, are being vigorously pursued to mitigate these limitations [73–75]. However, even in hematologic cancers, sustained therapeutic efficacy is often inadequate [76, 77]. This observation emphasizes the critical insight that the initial potency of an engineered immune response does not invariably translate into durable immunological control.

### The pervasive challenge of adaptive resistance to ICIs and ICT

Despite the unprecedented durability of responses achievable with cancer immunotherapy, the majority of patients exhibit either primary resistance (no initial response) or adaptive resistance (relapse after initial benefit) [78]. This pattern of relapse recurs across diverse therapeutic strategies and disease stages, implying that common biological underpinnings are rooted in immunoediting. Clinical data underscore this limitation, showing that while PD-1/PD-L1 blockade induces profound responses in a subset of patients, durable benefit is restricted to a minority of patients [79]. In melanoma, 40% to 65% of individuals fail to respond initially, and only 30% of initial responders maintain responses at 5 years because of adaptive resistance [80–82]. Comparable rates of adaptive resistance are observed in advanced NSCLC (e.g., more than 60% of initial responders to PD-1/PD-L1 ICIs develop adaptive resistance) and with CTLA-4 inhibition [83–85]. Crucially, empirical attempts to overcome this by combinatory treatment have often failed to improve overall survival, demonstrating that the barrier to adaptive resistance is not easily circumvented by simple combination strategies [86].

The emergence of neoadjuvant immunotherapy, while transformative, has not overcome this fundamental limitation. Although neoadjuvant pembrolizumab enhances pCR and event-free survival in early-stage NSCLC, not all patients achieve a complete response, and a proportion of initial responders still experience disease recurrence [87]. Even when administered prior to the establishment of a profoundly immunosuppressive TME, early intervention cannot guarantee durable immune memory. Management of irAEs also becomes particularly important in this curative-intent setting. The absence of reliable biomarkers for predicting a sustained response to neoadjuvant immunotherapy highlights fundamental gaps in the understanding of durable immune memory determinants. This adaptive resistance paradigm extends to ICT, where even optimally engineered T-cell products ultimately succumb to resistance mechanisms. In multiple myeloma, CD19-directed CAR-T-cell therapy induces initial complete responses in 73.0% of patients; however, subsequent disease progression occurs in the majority of patients, highlighting the persistent challenge of achieving sustained remission [76]. These findings collectively indicate that adaptive resistance manifests across both solid and hematologic malignancies, ultimately limiting the long-term efficacy of current immunotherapeutic strategies even in initial responders, as summarized in Table 1.

**Table 1:** Immunotherapy response and resistance profiles across tumors

Cancer type	Trial registration	Lines of therapy	Immunotherapeutic regimen	OS (months, HR 95% CI)	ORR/pCR (%)	Long-term response	Adaptive resistance rate	Remarks
**Melanoma**	NCT00094653	Second-line	A: Ipilimumab vs. gp100 vaccine; B: Ipilimumab + gp100 vaccine vs. Ipilimumab	A: 10.12 vs. 6.44, HR=0.66, *p*=0.0026 B: 9.95 vs. 10.12, HR=1.04, *p*=0.7575	ORR-A: 10.9 vs. 1.5 ORR-B: 5.7 vs 10.9	Ipilimumab 5-year OS rate 16%	30-50% among initial responders	First CTLA-4 inhibitor; durable response in a subset
**Melanoma**	CheckMate 067 (NCT01844505)	First-line	A: Nivolumab vs. Ipilimumab; B: Nivolumab + Ipilimumab vs. Ipilimumab	A: 36.93 vs. 19.94, HR=0.63, *p*<0.0001 B: 71.92 vs 19.94, HR=0.55, *p*<0.0001	ORR-A: 44.9 vs. 19.0 ORR-B: 58.3 vs. 19.0	2-year OS rate-A: 37% vs. 12% 2-year OS rate-B: 43% vs. 12%	NA	First dual immunotherapy to show long-term survival plateau
**Melanoma**	KEYNOTE-001 (NCT01295827)	First-line/Second-line	Pembrolizumab	NA	ORR 29.4-36.7	5-year OS rate 34%	25-35% among initial responders	Landmark 5-year survival data for anti-PD-1 monotherapy
**Melanoma**	CheckMate-066 (NCT01721772)	First-line	Nivolumab vs. Dacarbazine	NA vs. 10.84, HR=0.42, *p*<0.0001	ORR 42.4 vs. 14.4	1-year OS improved	NA	Nivolumab monotherapy improves survival vs. chemotherapy
**Melanoma**	From the Danish metastatic melanoma database	First-line/Second-line	Pembrolizumab vs. Placebo	NA	ORR 39.3	>20% with continued disease control	60% do not respond initially	Demonstrates anti-PD-1 efficacy in metastatic setting
**HCC**	Imbrave-150 (NCT03434379)	First-line	Atezolizumab + Bevacizumab vs. Sorafenib	19.22 vs. 13.4, HR=0.66, *p*=0.0009	ORR 27.3 vs. 11.9	Median OS 19.2 months	Majority progress	First-line standard in HCC
**UC**	Imvigor-210 (NCT02951767/NCT02108652)	Second-line	Atezolizumab	7.9-15.9	ORR 15.8-22.7	2-year OS rate 30%	High, especially in PD-L1^low^ tumors	First anti-PD-L1 agent approved in advanced UC
**NSCLC**	KEYNOTE-024/042/189	First-line	Pembrolizumab vs. Pembrolizumab + Chemo	13.4-30 (PD-L1-dependent)	ORR 27.3-47.6 (PD-L1-dependent)	5-year OS rate 20% PD-L1 >50%	>60% among initial responders	PD-L1^high^ tumors derive greatest benefit; KEYNOTE-189 established chemoimmunotherapy standard
**RCC**	CONTACT-03 (NCT04338269)	Second-line	Atezolizumab + Cabozantinib vs. Cabozantinib	25.72 vs. NA, HR=0.94, *p*=0.6902	ORR 38 vs. 41.7	No significant OS benefit	NA	Combination did not improve outcomes
**RCC**	CheckMate-214 (NCT02231749)	First-line	Nivolumab + Ipilimumab vs. Sunitinib	NA vs. 25.95, HR=0.63, *p*<0.0001	ORR 41.6 vs. 21.5	3-year OS rate 44%	Common after progression	Dual immunotherapy standard in advanced RCC
**R/R cHL**	KEYNOTE-087 (NCT02453594)	Second-line	Pembrolizumab vs. Placebo	NA	ORR 66.7-82.6	3-year PFS rate 60%	Lower than solid tumors, but still observed	High response rates in relapsed/refractory setting
**R/R ALL**	CART19 ClinicalTrials (NCT01626495/NCT01029366)	Second-line	CD19 CAR-T (Kymriah)	OS rate 78%	CR up to 90%	Durable remissions up to 24 months	NA	CTL019 was the first FDA-approved CAR-T therapy, which achieved high remission rate, but 27% patients developed severe cytokine-release syndrome
**R/R MM**	LEGEND-2 (NCT03090659/ChiCTRONH-17012285)	Second-line	LCAR-B38M CAR T cells (Cilta-cel)	Median follow-up of 65.4	CR 74%; MRD- up to 90%	5-year OS 49.1%	83.8% suffered PD and/or death; while 61.1% of PD patients responded to subsequent therapies	Cilta-cel was subsequently validated in the multicenter Phase III CARTITUDE-1 trial and has received marketing authorization from FDA and NMPA
**RCC**	KEYNOTE-564 (NCT03142334)	Adjuvant post-surgery	Pembrolizumab vs. Placebo	NA	NA	OS improvement vs. placebo	NA	First positive adjuvant immunotherapy trial in RCC
**NSCLC**	KEYNOTE-671 (NCT03425643)	Neoadjuvant	Pembrolizumab + Chemo vs. Chemo	NA vs. 52.4, HR=0.72, *p*=0.0052	pCR 18.1 vs. 4.0	5-year OS 64.6% vs. 53.6%	NA	First Phase III trial to show neoadjuvant immunotherapy plus chemotherapy followed by adjuvant immunotherapy can significantly improve both EFS and OS as dual endpoints
**TNBC**	KEYNOTE-522 (NCT03036488)	Neoadjuvant	Pembrolizumab + Chemo vs. Chemo	NA	pCR 64.8 vs. 51.2	3-year EFS rate 84.5%	Data maturing	First Phase III trial to show improved EFS with neoadjuvant immunotherapy in early-stage TNBC

*HCC* hepatocellular carcinoma, *UC* urothelial carcinoma, *NSCLC* non-small cell lung cancer, *RCC* renal cell carcinoma *R/R* relapsed or refractory, *cHL* classical hodgkin lymphoma, *ALL* acute lymphoblastic leukemia, *MM* multiple myeloma, *TNBC* triple-negative breast cancer, *Cilta-cel* ciltacabtagene autoleucel, *ORR* objective response rate, *pCR* pathological complete response, *EFS* event free survival, *OS* overall survival, *PFS* progression free survival, *DFS* disease free survival, *PD* progressive disease, *HR* hazard ratio, *NA* not available, *NMPA* National Medical Products Administration of China, *FDA* the U.S. Food and Drug Administration

### T-cell dysfunction as a bottleneck in cancer immunotherapy

While recent advances have enabled more precise targeting of resistance pathways, such as immune checkpoints, the suppressive tumor microenvironment, and antigen presentation defects, current immunotherapy strategies remain hampered by adaptive resistance, characterized by the consistent failure to sustain T-cell-driven antitumor responses over time. These recurrent therapeutic failures point to an intrinsic limitation in that the progressive dysfunction of tumor-specific T cells manifests as two interrelated defects, namely, insufficient effector function and the inability to establish durable AIM. We propose that this dual defect serves as a central mechanistic bottleneck underlying the long-term success of both ICI therapy and ICT. This framework is strongly corroborated by increasing clinical evidence. A significant proportion of patients who achieve initial tumor regression subsequently relapse, highlighting that cytoreduction is insufficient without the concomitant establishment of persistent immune surveillance. Conversely, patients who progress during treatment despite an initial response exemplify the consequences of insufficient effector function. Critically, emerging therapeutic strategies are now directly aimed at ameliorating these distinct T-cell deficits. For instance, a recent study demonstrated that poly(ADP-ribose) polymerase inhibition (PARPi) enhances the metabolic fitness of CD8^+^ T cells and generates therapeutically superior central memory CD8^+^ T cells via coordinated transcriptional and metabolic reprogramming, thus increasing the antitumor response [88]. Although such strategies are designed to enhance memory responses, clinical relapses still occur, underscoring that restoring memory alone may be insufficient if effector function is concurrently compromised. Evaluations of relapse timing, integrated with immune phenotyping data, further link early resistance to insufficient effector function and late-onset relapse to progressive loss of memory-sustaining T-cell subsets. Mechanistically, these divergent outcomes reflect fundamentally distinct T-cell differentiation trajectories. Patients who achieve durable clinical responses demonstrate coordinated maintenance of both effector-competent and memory-poised T-cell pools. This equilibrium is orchestrated by integrated transcriptional and epigenetic regulatory networks that sustain T-cell longevity while preserving immediate functional capacity. In resistant cases, by comparison, two distinct failure modes emerge, in which either terminally exhausted T-cell clones prevail and exhibit restricted stem-like potential and epigenetic fixation into dysfunctional states that impair immediate effector function or the memory precursor pool contracts, leading to loss of long-term immune surveillance. Collectively, these findings refine our understanding by revealing that the central challenge lies not only in initiating an antitumor response but also in ensuring its bifurcated differentiation into robust effector cells and durable, self-sustaining memory populations. Failure to properly establish or maintain either population creates a permissive environment for immune escape. Consequently, this dual-defect framework provides a unified explanation for how on-treatment progression and postremission relapse both stem from progressive T-cell dysfunction. The following sections explore how immunoediting disrupts these critical T-cell differentiation trajectories.

## Mechanisms of adaptive resistance to cancer immunotherapy

**Fig. 2:**
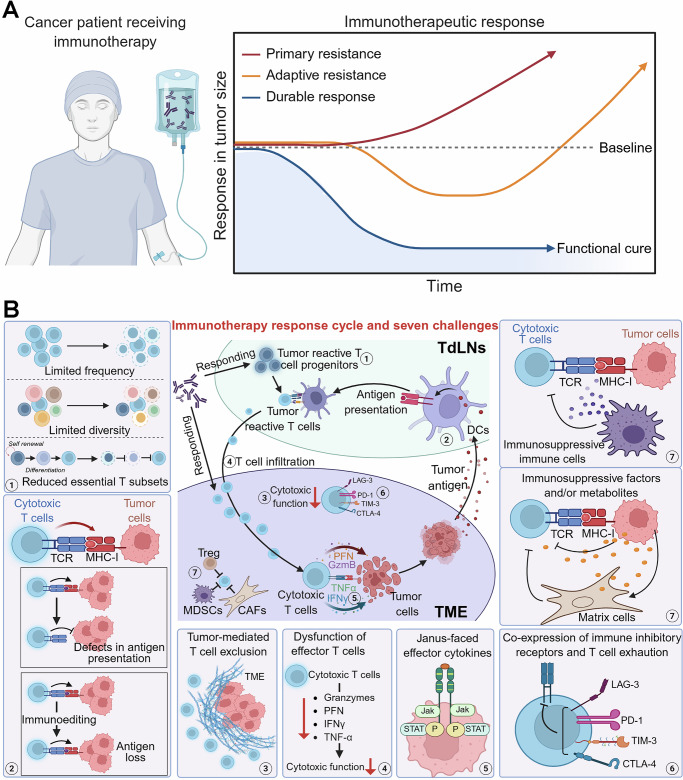
Clinical trajectories of adaptive resistance and the seven challenges in the immunotherapy response cycle. This integrated figure connects the conceptual framework of antitumor immunity with its clinical manifestation. The upper graph (**A**) illustrates three distinct clinical trajectories following immunotherapy, with a particular focus on adaptive resistance—a pattern in which an initial response is followed by disease relapse (orange curve). For comparison, the red curve represents primary resistance, defined as progressive tumor burden without an initial response, while the blue curve represents a durable response, leading to long-term tumor control and functional cure. Together, these trajectories capture the spectrum of clinical outcomes, with adaptive resistance representing a critical challenge to treatment durability. These clinical patterns are driven by the underlying resistance mechanisms shown in the lower panel (**B**), which directly or indirectly compromise T-cell-mediated antitumor immunity. To systematically understand how adaptive resistance arises, we categorized these mechanisms into seven challenges during the immunotherapy response cycle: ① Lack of tumor-specific T-cell clonotypes and essential T-cell subsets, especially the precursor CD8^+^ T cells residing in the TME and TdLN. ② Defects in tumor antigen loss or presentation. ③ Tumor-mediated T-cell exclusion. ④ Dysfunction of effector T cells. ⑤ Janus-faced effector cytokines. ⑥ Coexpression of inhibitory immune checkpoints (e.g., PD-1, CTLA-4, LAG-3, and TIM-3) and T-cell exhaustion. ⑦ T-cell function is suppressed by immunosuppressive cells, secreted factors, and/or metabolites

As we mentioned above, resistance to cancer immunotherapy can be classified into primary resistance and adaptive resistance according to clinical outcomes [78]. Primary resistance refers to patients who do not respond at all to immunotherapy, while adaptive resistance refers to patients who have a period of initial response followed by disease progression (Fig. 2, panel A). Currently, the combination of immunotherapy with radiotherapy, chemotherapy, or targeted therapy has shown some potential in addressing this de novo resistance, yet approved strategies to counteract adaptive resistance remain limited, partly owing to highly heterogeneous treatment responses across tumors [89–91]. This challenge has brought adaptive resistance, a more dynamic and flexible form of resistance shaped by continuous tumor–immune interactions, to the forefront of current cancer research. Adaptive resistance is a complex process in which tumors actively remodel both their intrinsic properties and the microenvironment to evade immune attack under persistent immunotherapeutic pressure [92]. This phenomenon epitomizes Darwinian evolution, as immunotherapy exerts selective pressure that favors the expansion of tumor cell clones with novel immune evasion mechanisms. Thus, adaptive resistance can be viewed as the evolutionary response of the tumor ecosystem to therapeutic stress [92, 93]. A central consequence of this evolution is the failure to establish or sustain a durable AIM, leading to relapse despite the initial response.

To systematically understand tumor evolution toward adaptive resistance following immunotherapy, this section employs the cancer-immunity cycle framework, a concept proposed by Ira Mellman and Daniel S. Chen in 2013 and refined in 2023, to systematically elaborate the adaptive resistance mechanisms in cancer immunotherapy, including impaired antigen presentation, remodeling of the immunosuppressive TME, and upregulation of inhibitory ligands and receptors, among others. Such a structured approach enables more accurate prediction of immunotherapeutic resistance and guides the rational design of interventions to overcome it [94, 95]. The cancer-immunity cycle can be divided into several sequential steps as follows: tumor antigen release and presentation; T-cell priming and activation; tumor trafficking and infiltration; tumor recognition and killing; and the formation and maintenance of AIM [95]. This framework is valuable for identifying precisely where the antitumor immune response succeeds or fails. During this cycle, CD8^+^ T cells are pivotal front-line effector cells responsible for ultimately eliminating tumor cells. However, under chronic antigen exposure, these initially activated CD8^+^ T cells progressively adopt an exhausted phenotype, marked by hierarchical loss of effector functions and memory epigenetic profiles, along with upregulated inhibitory receptors such as PD-1 and CTLA-4, thereby impeding the final steps of the cycle [96, 97]. In cancer immunotherapy, particularly ICIs and ICT, these exhausted T cells should be reinvigorated. In therapeutic-responsive patients, tumors show increased infiltration by cytotoxic CD8^+^ T cells with low TCRβ diversity, which is indicative of the clonal expansion of tumor-specific T cells [32]. Consequently, when some initially responding patients develop resistance, the cancer-immunity cycle guides us to examine which specific stage (e.g., T-cell priming, trafficking and infiltration, or ultimately killing) is compromised, thereby impairing the CD8^+^ T-cell-mediated antitumor response. Targeting these defective steps through rational combination therapies holds promise for resensitizing patients who are resistant to immunotherapy.

Accordingly, we organize the reported adaptive resistance mechanisms on the basis of the cancer–immunity cycle in which they are disrupted, aligning them with our conceptual framework. The mechanisms are broadly categorized as follows (Fig. 2, panel B): **(1)** failures in T-cell activation and priming (Fig. 2, panels B-①), such as a lack of tumor-specific T-cell clonotypes and essential T-cell subsets, especially the precursor exhausted CD8^+^ T cells (Tpex) within the TME and tumor-specific memory T cells (T_TSM_) residing in the TdLN (TdLN-T_TSM_). These populations are central to the AIM, which sustains durable clinical responses. They are major responders to ICI therapy and promising candidates for ICT, and their loss reflects an adaptive evasion mechanism during the equilibrium and escape phases of immunoediting. **(2)** Impaired initial antitumor immunity (Fig. 2 panel B-②): defects in tumor antigen loss/presentation. This represents a failure in the elimination phase of immunoediting, allowing tumors to escape initial immune recognition by disrupting neoantigen presentation and preventing the activation of naïve T cells. **(3)** Barriers to T-cell trafficking and infiltration (Fig. 2 panel B-③): tumor-mediated T-cell exclusion. By physically or functionally blocking T-cell entry into the TME, tumors enforce an immune-privileged niche, a hallmark of the escape phase that prevents effector T cells from reaching and engaging tumor cells. **(4)** Suppression of T-cell function within the TME (Fig. 2 panel B-④~⑦): This involves dysfunction of effector T cells, T-cell exhaustion, coexpression of PD-1/CTLA-4 with other inhibitory immune checkpoints, context-dependent dual roles of interferon (IFN) signaling in both stimulatory and suppressive antitumor immunity, and T-cell death as a barrier to sustained antitumor immunity. Collectively, these mechanisms undermine T-cell-mediated antitumor immunity, promoting immune evasion and tumor progression. They also impair the differentiation and functional maturation of antitumor memory T cells, thereby limiting the durability of immunotherapeutic responses.

### Impaired initial antitumor immunity

Defects in the initial steps of antitumor immunity, including tumor antigen availability, processing, and presentation, represent a fundamental barrier to an effective response to cancer immunotherapy, as they prevent the immune system from even recognizing tumor cells as targets. This process is primarily orchestrated through a sequential cascade as follows: tumor-generated immunogenic antigens are captured and presented by antigen-presenting cells (APCs) via MHC molecules, which display antigenic peptides to naïve T cells, subsequently inducing T-cell priming. The most direct impairment involves the downregulation or complete loss of components essential for the MHC class I (MHC-I) presentation pathway, including the MHC-I molecules themselves, transporters associated with antigen processing (TAP1/TAP2), and β2-microglobulin (B2M). This effectively renders tumor cells invisible to CD8^+^ T cells. Beyond the presentation machinery, the source of the antigens themselves can be problematic. Tumors with a low tumor mutational burden (TMB) generate few neoantigens, or they undergo antigen-loss variants under immune pressure. Furthermore, impaired recruitment, activation, or function of APCs, particularly dendritic cells (DCs), disrupts the critical cross-priming of tumor-specific CD8^+^ T cells in the lymph nodes. Collectively, these impairments highlight that the initiation of an antitumor immune response is a fragile and multistep process that relies on the seamless progression from tumor antigen generation to successful T-cell priming. It is therefore unsurprising that disruptions at any point within this sequence can lead to adaptive resistance,  dooming immunotherapy from the outset.

We take adaptive resistance to anti-PD therapy as an example. Among patients who initially respond to anti-PD therapy, the renaissance of antitumor immunity might lead to the selection of tumor clones with the capacity to subvert T-cell responses. Samples from 44 individuals with melanoma following anti-PD-1 treatment were subjected to clustered regularly interspaced short palindromic repeats (CRISPR) screenings of tumor cells escaping PD-1 blockade, and approximately 25% of the resistant tumors exhibited genetic alterations in B2M and the janus tyrosine kinase 1/2-signal transducer and activator of transcription (JAK1/2-STAT) signaling pathways [98]. These mutations in cancer cells result in genetic deficiency in antigen presentation via MHC-I, as well as increased PD-L1 expression and decreased responsiveness to IFN-γ. Patients harboring such tumors grow increasingly resistant to anti-PD-1 antibodies during treatment [98, 99]. Additionally, whole-exome sequencing of NSCLC patients treated with pembrolizumab revealed that the durable clinical efficacy of pembrolizumab strongly correlated with increased mutation and neoantigen burden [100]. Another recent cohort of 80 patients with NSCLC treated with or without nivolumab also demonstrated that early loss of mutations and neoantigens during anti-PD therapy is negatively associated with clinical benefit [101]. These clinical trials imply that tumors (e.g., NSCLC) with high mutational loads are susceptible to anti-PD therapy [102], suggesting that the pathways associated with tumor antigen loss may lead to adaptive anti-PD resistance. Indeed, studies of tumors with poor immunogenicity (e.g., pancreas or prostate) have demonstrated that insufficient tumor antigens prime suboptimal or poor antitumor immunity, likely rendering ineffective anti-PD therapy. Consequently, neoantigen immunoediting may drive anti-PD therapy efficacy [3, 101], and the tumor mutational burden has been shown to have predictive potential for identifying responders and nonresponders prior to or during treatment [103, 104].

On the other hand, even if tumor antigens can be generated, the impaired recruitment and function of APCs, particularly DCs, constitute a third major barrier to initial antitumor immunity. As key professional APCs, DCs are indispensable for capturing tumor antigens, migrating to draining lymph nodes, and cross-presenting them to prime naïve CD8^+^ T cells. The importance of DCs has been highlighted by several studies, which indicate that their absence or dysfunction within the tumor bed is a primary factor contributing to resistance to cancer immunotherapy. This impairment occurs as the TME actively disrupts the DC lifecycle at multiple levels. First, inadequate chemokines and the presence of barriers can cripple the initial recruitment of DC precursors to the tumor bed. DC accumulation in tumors often depends on the DC chemoattractants CCL5, XCL1, and XCL2 produced by natural killer (NK) cells [105]. As a result, tumors impair NK cell viability and chemokine production through the secretion of prostaglandin E2 (PGE2) to evade the NK cell-DC axis and immunotherapy. Some ICI-resistant tumors inhibit the recruitment and migration of DCs harboring tumor antigens by downregulating the expression of some chemokines, such as CCL4 and CCR7 [106, 107], ultimately contributing to ICI resistance. The tumor-derived cellular regulatory factors PGE2 and transforming growth factor-β (TGF-β) upregulate PD-L1 expression by DCs, turning them into immunosuppressive cells [108]. Tumors can also utilize myeloid plasticity to directly manipulate dendritic cell differentiation, skewing it toward immune-suppressive macrophage-like subsets that correlate negatively with the immune response to anti-PD-L1 and anti-CTLA-4 therapies [106]. Furthermore, tumor cells produce high levels of lactic acid, which suppresses interleukin-12 (IL-12) production and inhibits DC-dependent antigen presentation via G protein-coupled receptor 81 (GPR81) signaling, the specific receptor of lactic acid [109]. Tumors can also hijack DC maturation to decrease MHC expression and destroy the costimulatory machinery by secreting suppressive factors such as TGF-β, interleukin-10 (IL-10), and vascular endothelial growth factor (VEGF) [110]. The absence of effective costimulatory signals potentially renders immature DCs tolerogenic and incapable of initiating an immune response. A recent study revealed that combining ICI therapy with manganese or MSA-2 (a stimulator of interferon gene agonists) synergistically increased antitumor efficacy and rescued adaptive resistance by promoting DC maturation and tumor-specific antigen presentation [111, 112].

However, other myeloid cells within the TME, particularly macrophages, also express antigen-presenting machinery. Nevertheless, their relatively lower efficiency than that of DCs generally leads to T-cell suppression rather than activation. For instance, tumor-associated macrophages (TAMs) exert a dominant inhibitory effect by significantly upregulating the expression of coinhibitory ligands such as PD-L1, thereby reducing the proportion of activated CD8^+^ T cells, suppressing their proliferation, and potentially inducing clonal anergy or deletion [113, 114]. A recent study of hepatocellular carcinoma published by Kuang’s laboratory revealed that pericancerous macrophages cross-present antigens to CD103^+^ cytotoxic T lymphocytes to result in their retention in the pericancerous area, thus inducing the activity of cytotoxic T cells to exert protumorigenic effects and resistance to PD-1-directed immunotherapy [115]. Additionally, some tumor-resident T-cell clusters, such as an endothelial cluster from ovalbumin (OVA)-overexpressing B16 (B16-OVA) melanoma, can function as APCs to potentially regulate therapeutic efficacy by impacting antigen presentation. These nonprofessional APCs expressing PD-L1 and MHC class II (MHC-II) can take up and present tumor-derived peptides and inhibit OVA-specific CD8^+^ T-cell proliferation and cytotoxicity via the PD-1/PD-L1 pathway, as well as induce immunosuppressive CD4^+^ T cells in an antigen-specific manner [116].

While defective DC function undermines the priming of a tumor-specific T-cell response, the efficacy of even preprimed or engineered T cells such as those used in ICT can be thwarted at the level of target recognition. The problems of antigen loss and impaired presentation pose equally fatal challenges. This impairment constitutes a fundamental barrier across multiple immunotherapy modalities, as it serves the critical link between the immune effector and its target antigen. This failure in target recognition manifests differently but converges on the same outcome. For CAR-T cells, which recognize surface antigens, the mere downregulation or loss of the target antigen (a phenomenon known as antigen escape) is sufficient to confer resistance [117]. With respect to TCR-T cells, which, like natural T cells, recognize intracellular antigens presented by MHC molecules, any disruption in antigen processing and presentation pathways, such as mutations in the MHC itself or components such as B2M, can lead to complete immune evasion [118]. Consequently, whether through the failure to initiate an immune response via DCs or the failure to execute one via antigen/presentation loss, the impairment of this initial phase represents a foundational axis of resistance across the immunotherapy landscape. Indeed, even if downstream T-cell function is intact, the absence of proper antigen recognition dooms  cancer immunotherapy to fail.  This brings  us to the next critical barrier: failures in T-cell activation and priming.

### Failures in T-cell activation and priming

A robust antitumor T-cell response is fundamental to adaptive immunity. Defective T-cell activation and priming undermine this response and can directly contribute to the lack of tumor-reactive T-cell clones. Therefore, even when various immunotherapies are applied to alleviate T-cell suppression, the treatment fails because of the absence of target cells. First, for ICI treatment, previous studies in melanoma and colorectal tumors have established that tumor regression following therapeutic PD-1 blockade requires preexisting tumor-specific CD8^+^ T cells [32, 119]. These cells are negatively regulated by PD-1/PD-L1-mediated adaptive immune resistance, highlighting that the presence of tumor-specific CD8^+^ T cells is not only a predictor of response but also a key determinant of sensitivity to anti-PD treatment. The absence of tumor-reactive T-cell clones likewise compromises ICT, which fundamentally relies on the isolation and expansion of these specific cells from tumor tissue or peripheral blood [120]. Furthermore, in solid tumors, the efficacy and persistence of CAR-T cells are constrained by a host T-cell environment that, when devoid of endogenous tumor-specific clones, may fail to support the sustained function of the infused products [121, 122]. Thus, the lack of tumor-reactive clones constitutes a common limitation across multiple immunotherapy modalities. As a result, the factors resulting in a scarcity of tumor-specific CD8^+^ T cells within tumors presumably restrict the response to cancer immunotherapies. This scarcity may stem not only from insufficient priming but also from the functional inactivation of existing clones. Effective T-cell activation requires not only the presence of a cognate antigen but also its proper presentation. However, tumors often exhibit poor immunogenicity or actively suppress effective antigen presentation. On the one hand, DCs within the TME frequently display an immature or tolerogenic phenotype, characterized by reduced expression of costimulatory molecules and impaired cross-presentation capacity—limitations that hinder their ability to prime naïve T cells, as discussed earlier [123, 124]. On the other hand, tumor cells themselves may downregulate MHC-I molecules or components of the antigen-processing machinery, thereby diminishing recognition and cytotoxicity by CD8^+^ T cells and weakening the antitumor efficacy of immunotherapies aimed at restoring CD8^+^ T-cell function [125]. Beyond defects in antigen presentation, the harsh TME profoundly suppresses T-cell activation through a variety of cellular and soluble mediators. The TME encompasses numerous immunosuppressive cell types, such as cancer-associated fibroblasts (CAFs), TAMs, myeloid-derived suppressor cells (MDSCs) and Foxp3^+^ regulatory CD4^+^ T cells (Tregs). These cell populations impose metabolic constraints (e.g., glucose deprivation) and secrete inhibitory factors that directly disrupt TCR signaling and costimulatory pathways, which are essential for optimal T-cell activation. This in turn induces dysfunction of tumor-specific CD8^+^ T cells and contributes to adaptive resistance to immunotherapies [126–130]. For instance, Tregs consume interleukin-2 (IL-2) and produce TGF-β, thereby depriving effector T cells of critical survival and proliferation signals during the activation process [131]. MDSCs generate reactive oxygen species (ROS) and deplete essential amino acids, creating a metabolically restrictive environment that places T cells in a quiescent state [132, 133]. Superimposed on this cellular landscape is a complex array of soluble immunosuppressive factors that further impair T-cell activation. The TME harbors elevated levels of immunosuppressive cytokines, including interleukin-4 (IL-4), interleukin-6 (IL-6), IL-10, and TGF-β, which can directly blunt TCR signaling and inhibit the expression of cytolytic molecules [134–139]. Metabolic byproducts such as adenosine, generated via CD39 and CD73 on tumor and stromal cells, engage A2A receptors on T cells, increasing intracellular cAMP levels and suppressing early TCR signaling events [140]. Similarly, tryptophan catabolism by indoleamine 2,3-dioxygenase (IDO) produces kynurenines that promote T-cell anergy and apoptosis [141–143]. Together, these soluble mediators create a biochemical environment inherently hostile to the initiation and maintenance of effective T-cell activation, even in the presence of cognate antigen and costimulatory signals. However, even when sufficient tumor-specific T-cell clones are present, their functional state and differentiation fate within the immunosuppressive TME are equally critical, as an unsustainable immune response ultimately leads to treatment failure. In the TME, functionally suppressed T cells are predominantly exhausted and exhibit significant heterogeneity. In addition to cell-intrinsic exhaustion programs, the successful infiltration of activated T cells into the tumor bed is a dynamic, multistep process that can be conceptualized as a two-stage activation cascade. Kissick’s laboratory reported for the first time that tumor-specific CD8^+^ T cells were initially activated in TdLNs to acquire a stem-like phenotype with self-renewal and proliferative potential. These CD8^+^ T cells subsequently migrate to the tumor bed in a stem-like state, where they undergo further restimulation within the TME and develop a canonical effector program to unleash cytotoxicity upon encountering tumor cells [144]. Their work highlights the crucial role of the TdLN priming phase in shaping the ultimate antitumor efficacy and offers a new perspective for understanding why certain TMEs fail to support efficient T-cell differentiation. Accordingly, any factors that restrict the migration of these stem-like CD8^+^ T cells from TdLNs into tumor sites presumably constitute a critical barrier for resistance to cancer immunotherapy, including dysregulated chemokine signaling, vascular barriers, and physical stromal obstacles. Having delineated the core barriers that lead to the numerical paucity of tumor-specific T cells in the TME, we now turn to a contrasting layer of complexity: the numerical dominance of tumor-irrelevant bystander T cells. In 2018, the Newell Laboratory performed sequencing analysis on TILs from a variety of human cancers and reported that the majority of CD8⁺ T cells within tumors are not tumor antigen-specific but instead consist of bystander T cells that recognize unrelated antigens, such as those derived from viruses. The proposal and subsequent validation of this concept fundamentally reshaped the understanding of T-cell repertoire composition in tumors, prompting a fundamental reassessment of the mechanisms underlying T-cell-mediated immunosurveillance and the precise targets of tumor immunotherapy. Despite their antigenic irrelevance, these cells not only are abundant within the TME but also frequently retain memory-associated properties and functional vigor [145–147]. In addition, bystanders, including those with specificity for viral pathogens, typically lack expression of canonical exhausted markers (e.g., PD-1, CD39, and CXCL13) and exhibit low tumor reactivity signatures [146, 148, 149]. Rather than being inert bystanders, they can actively sculpt the functional landscape of the TME. Our group recently demonstrated that redirecting the cytotoxic potential of these preexisting functional bystander T cells toward tumor cells using an engineered oncolytic virus could largely curtail tumor growth and synergistically improve the efficacy of anti-PD-L1 therapy across multiple preclinical tumor models [150]. Hence, although tumor-reactive T cells are numerically insufficient and functionally debilitated in the TME, strategically modulating preexisting functionally competent bystander T cells may offer a previously underappreciated avenue for overcoming the current limitations of cancer immunotherapy in the future. Collectively, these results underscore that both the abundance and the spatiotemporal dynamics of activated T cells within the TME are critical determinants of the development of adaptive resistance to immunotherapies. However, the establishment of such an effective antitumor immune niche presupposes the successful recruitment of T cells into the tumor parenchyma. We next discuss the barriers that prevent T-cell infiltration into the tumor bed and contribute to therapeutic resistance.

### Barriers to T-cell trafficking and infiltration

Once activated, T cells migrate from the bloodstream and infiltrate the tumor parenchyma, a rate-limiting step in the cancer-immunity cycle [94]. Under physiological conditions, T-cell trafficking and infiltration proceed through a multistep cascade comprising tethering, rolling, arrest, transendothelial migration, and chemokine-guided interstitial navigation. However, tumors actively impede this process by dynamically remodeling their microenvironment and creating multiple barriers that drive adaptive immunotherapy resistance [151]. Abnormal tumor vasculature constitutes the first physical barrier to T-cell extravasation [152]. Vessels are often disorganized, tortuous, and hyperpermeable, leading to heterogeneous blood flow that prevents stable T-cell‒endothelial contact [153]. Moreover, the expression of adhesion molecules such as intercellular adhesion molecule 1 (ICAM-1) and vascular cell adhesion molecule 1 (VCAM-1) is frequently downregulated, whereas the expression of immunosuppressive ligands such as PD-L1 is often upregulated, collectively impairing T-cell adhesion and transmigration. This vascular abnormalization is not a passive defect but an active adaptation of the tumor under immune selection pressure. By obscuring molecular signs and deploying immune checkpoints, tumors effectively transform a potential route for T-cell entry into a frontline defense against immune attack. Beyond the vessels, T cells must navigate the tumor stroma to reach cancer cell nests, a process impeded by a second major barrier. Activated CAFs overproduce and cross-link extracellular matrix (ECM) components, creating a fibrotic constraint that physically blocks T-cell movement [154]. Elevated interstitial fluid pressure further constrains the space available for T-cell movement. CAFs and tumor cells secrete factors such as TGF-β and CXCL12, which suppress T-cell effector function and sequester T cells within the stromal compartment [155, 156]. Thus, the stroma acts as a dynamically remodeled shield that physically excludes and chemically disorients infiltrating T cells. Throughout this process, T cells also encounter immunosuppressive cells that form a pervasive cellular barrier. MDSCs often accumulate near the vasculature, depleting nutrients such as L-arginine and releasing mediators that impair T-cell adhesion and cytotoxicity [157, 158]. Tregs are preferentially recruited to tumor beds, where they establish a potent immunosuppressive milieu through the secretion of IL-10 and TGF-β, as well as by competing for IL-2 [159]. By coopting host immune cells, tumors actively harass, deplete, and suppress T cells, ultimately dismantling their cytotoxic potential.

As summarized above, the dense ECM barrier and abnormal vasculature, together with immunosuppressive immune cells, cooperatively establish an immune‑privileged TME. This niche systematically excludes T cells and inhibits their function, thereby enabling tumors to evade immune destruction independent of antigenic mutations. Such a mechanism underlies broad adaptive resistance to multiple forms of immunotherapy, and resistance to PD‑1/PD‑L1 blockade provides a well-characterized paradigm for understanding this escape mechanism. A T-cell-exclusive program expressed by tumor cells prior to immunotherapy contributes to immunotherapeutic resistance, which is applicable to other T-cell-directed therapies as well [160, 161]. In contrast, the Ribas laboratory revealed that in melanoma patients who respond well to anti-PD-1 treatment, the proliferation of intratumoral CD8^+^ T cells is directly correlated with a reduction in tumor size [32]. Importantly, pretreatment samples obtained from responding patients show increased numbers of CD8-, PD-1-, and PD-L1-expressing cells within tumors. A subsequent study of advanced solid malignant tumors by the Ferté laboratory developed a radiomic signature for CD8^+^ T cells to estimate CD8^+^ T-cell counts in the TME and concluded that in patients treated with anti-PD-1 therapy and anti-PD-L1 therapy, a high baseline radiomic score was associated with a high objective response rate (ORR) at 3 months [162]. These findings reveal that the therapeutic benefit of PD1/PD-L1 blockade is highly dependent on preexisting T-cell infiltration, a finding that was subsequently confirmed to be a common basis for the responses to various immunotherapies, such as CTLA-4 blockade and ICT treatment [106]. As a result, a pretreatment TME rich in antigen-experienced, proliferating CD8⁺ T cells (e.g., CD8⁺CD45RO⁺Ki67⁺) is considered a predictive biomarker for immunotherapy response [163, 164].

On the basis of the above studies, preexisting T cells within the TME are considered pharmacodynamic or predictive markers. However, this paradigm, in which immunotherapy works primarily by rebooting preexisting activated exhausted tumor-infiltrating CD8^+^ T cells [165], has been challenged by several recently published studies. The Chang laboratory demonstrated that although TCF1-positive tumor-specific T cells were more likely to persist and proliferate in the tumor area after anti-PD-1 treatment, only a minority of the expanded TCR clones arose from TCF1-positive cells [166]. The majority (84%) of these exhausted T cells had novel TCRs that were not identified in the tumor but were detected in the peripheral blood before therapy. These findings, which are consistent with our observations [167], support the new hypothesis that the response to PD-1 blockade relies on the intrinsic ability of tumors to newly recruit T cells with novel TCR specificities from either peripheral blood or TdLNs, which replaced preexisting exhausted T cells within tumors that might have a limited capacity to reinvigorate the anti-PD response. These findings redefine our understanding of T-cell engagement during immunotherapy, shifting the focus from solely reinvigorating exhausted T cells to the pivotal process of recruiting new T cells, a principle that is likely universally applicable to T-cell-centric regimens. By implication, the durable efficacy of cancer immunotherapy is limited by factors, both tumor intrinsic and extrinsic, that restrict T-cell migration from the source(s) into the tumor regions (e.g., with deficits in T-cell-attracting chemokines, including CCL5 and CXCL9, -10, and -11/CXCR3 axis) and infiltration posttherapy [168–170]. Consequently, the failure of T-cell infiltration, which results from the multitiered barriers imposed by tumors, serves as a foundational and broadly adaptive resistance mechanism that directly impedes the clinical outcomes of multiple T-cell-centric immunotherapies. Nevertheless, even upon successfully overcoming these barriers, the antitumor capacity of T cells is further subverted by some deeper, functionally suppressive mechanisms inherent to the TME, constituting a formidable second line of defense in the adaptive resistance repertoire.

First, the spatial distribution of T cells is as critical as their absolute quantity. Even when T cells successfully enter tumor areas, their spatial features, such as their location within the TME, distribution, clustering, infiltration patterns, and topographical heterogeneity, can influence their response to treatment and immunotherapy outcomes and might guide therapeutic avenues [171–176]. In 2006, Galon and his colleagues introduced the concept of the Immunoscore on the basis of systematic immunohistochemical analysis of a large cohort of human colorectal cancer tissue samples. This paradigm uses the density and spatial distribution of CD3⁺ and CD8⁺ T cells within the tumor core and invasive margin as stronger prognostic indicators than the conventional tumor node metastasis staging system does [119]. Subsequently, a study from the Galon laboratory systematically mapped, for the first time, the spatial distribution of different immune cell subsets and their interrelationships within tumors across various cancer types using multiplex immunofluorescence and spatial analysis, significantly advancing the immune score framework into the era of high‑dimensional spatial phenotyping [177]. Their work demonstrated that quantifying and mapping TILs has significant clinical value, not only spurring the clinical adoption of the Immunoscore as a promising predictor biomarker for therapeutic efficacy but also highlighting a pivotal advance in transforming TME assessment from descriptive observation to a quantitative prognostic framework. Taking melanoma as an example, the presence of T cells within the invasive margin, but not necessarily the tumor parenchyma, was correlated with sensitivity to anti-PD-1 therapy [178, 179]. These findings indicate that the infiltration of T cells at the tumor invasion margin may be an important indicator for predicting the response to PD-1 blockade. Further study revealed that in both anti-PD-1-alone and anti-PD-1 plus ipilimumab-treated patients, compared with nonresponders, responders had markedly greater densities of CD8^+^ T cells in proximity to PD-L1-expressing cells (e.g., melanoma cells), which was associated with enhanced progression-free survival (PFS) for both therapeutic approaches [180, 181]. These findings consistently suggest that the spatial score of infiltrated T cells is an important marker for predicting the response to T-cell-centric therapies. Second, immunosuppressive myeloid cells represent a major barrier contributing to therapeutic resistance. Some tumors are refractory to ICIs, partly because of the presence of immunosuppressive myeloid cells in tumors. For instance, a population of macrophages with high levels of SPP1 transcripts has been identified in tumors from patients with advanced metastatic castration-resistant prostate cancer (mCRPC), which suppressed CD8^+^ T-cell activity and promoted PD-1 resistance [182]. Notably, tumor-induced expression of PD-L1 is largely limited to some highly immunosuppressive cells, such as pan macrophages and Ly-6C^+^ MDSCs [183]. As such, the generally low PD-L1 expression within prostate tumors not only underlies the low response to anti-PD therapy but also partially explains why certain cancer types exhibit broad insensitivity to immunotherapy [184]. Moreover, these PD-L1^+^ cells, which also express high levels of cyclooxygenase-2 (COX2) and PGE2, exhibit immunosuppressive properties and can eliminate CD8^+^ T cells, thereby contributing to adaptive resistance to pembrolizumab treatment [185, 186]. Inhibition of the COX2-PGE2 axis increases immune cell infiltration within the TME and rescues cytotoxic CD8^+^ T-cell activity, thus suppressing tumors by resensitizing pembrolizumab-resistant tumors [183]. These findings establish certain myeloid subsets as negative regulators of T-cell function, highlighting their potential as both broad-spectrum therapeutic targets and predictors of immunotherapeutic efficacy. Finally, multiple molecular pathways are actively involved in orchestrating T-cell exclusion. One anti-PD-resistant lesion harbors a unique immunological niche with nonexpanded T-cell clones, and such immune-poor microenvironments are devoid of PD-1^+^ CD8^+^ T cells, indicating that the suitable infiltration of CD8^+^ T cells is a prerequisite for tumor regression after therapeutic PD-1 blockade [98]. High activity of TGF-β [187, 188], histone deacetylase 8 (HDAC8) [189], p21-activated kinase 4 (PAK4) [190, 191], protein phosphatase 2 A (PP2A) [192], cyclin-dependent kinase 4/6 (CDK4/6) [160, 193], orphan GPR182 [194], and WNT/β-catenin [195–197], as well as loss of phosphatase and tensin homolog (PTEN) [198–200], hamper CD8^+^ T-cell infiltration and promote their exclusion. All of the above factors that induce CD8^+^ T-cell exclusion contribute to an immunosuppressive TME, which represents a fundamental mechanism underlying most immunological “cold” tumors that are unresponsive to anti-PD treatment and other forms of immunotherapy [187].

Given the critical role of T-cell infiltration in determining responses to immunotherapy, substantial efforts have been made to overcome the abovementioned barriers. These strategies can be broadly classified into those targeting vascular abnormalities, chemokine networks, and physical stromal obstacles. The tumor vasculature is characterized by structural and functional abnormalities, including tortuous and leaky vessels that impede efficient T-cell entry. Vascular normalization strategies aim to restore vessel integrity and improve perfusion. Anti-angiogenic agents, particularly anti-VEGF antibodies (e.g., bevacizumab) and tyrosine kinase inhibitors (e.g., axitinib), have been shown in preclinical models to transiently normalize tumor vasculature and promote T-cell infiltration [201, 202]. Emerging approaches include the use of angiopoietin-2 (ANGPT2) inhibitors, which stabilize vessels and augment T-cell infiltration. ANGPT2 blockade has been shown to promote CD8⁺ T-cell recruitment to tumor cores and improve vascular integrity [203, 204]. Beyond targeting the vasculature, restoring disrupted chemokine gradients represents another promising avenue. Preclinical studies have shown that the intratumoral delivery of T-cell-attracting chemokines (e.g., CXCL9) can increase T-cell infiltration. Epigenetic modulation offers an alternative approach, as DNA methyltransferase and histone deacetylase inhibitors can derepress silenced CXCL9 and CXCL10 genes in tumor cells, thereby increasing effector T-cell infiltration [205]. Additional approaches include conventional therapies (e.g., radiotherapy and chemotherapy) that induce chemokine upregulation, as well as oncolytic viruses engineered to express chemokines [206–208]. Notably, the intratumoral administration of IFN-γ increases chemokine production but may fail to enhance infiltration when other barriers persist, highlighting the need for multipronged combination strategies [209]. The dense ECM and CAFs that serve as physical barriers to T-cell penetration have also become therapeutic targets. CAFs can be targeted through fibroblast activation protein (FAP)-directed therapies, including FAP-targeted antibodies and FAP-CAR‑T cells. Preclinical studies have shown that FAP-CAR‑T cells effectively deplete CAFs, reduce fibrosis, and increase T-cell infiltration [210]. In contrast, agents directly targeting the ECM, such as PEGylated hyaluronidase (PEGPH20), have shown limited efficacy as monotherapies in patients with hyaluronan-high metastatic pancreatic cancer, prompting ongoing efforts to optimize combination strategies that address multiple barriers simultaneously [211]. For example, combining anti-VEGF therapy to normalize the vasculature, chemokine pathway agonists to enhance chemotaxis, and FAP-targeted agents to remodel stromal barriers has demonstrated synergistic effects [212]. These combinatorial strategies aim to create a permissive TME that supports robust T-cell infiltration, thereby overcoming  a foundational bottleneck to effective antitumor immunity. The entry‑promoting strategies outlined above are designed to achieve this initial step, and their  rational integration with immunotherapies acting at later stages is discussed in the section “*Emerging therapeutic strategies to overcome adaptive resistance*”.

In summary, tumors develop a coordinated, multilayered network of adaptive resistance, ranging from primary barriers to T-cell infiltration to secondary defenses that disrupt their spatial distribution and intrinsic function. This network underlies both immune privilege and the immunosuppressive TME, posing a common obstacle to various immunotherapies. Therefore, the fundamental strategy for overcoming adaptive immunotherapeutic resistance lies in systematically dismantling this integrated system. This involves not only targeting specific pathways to enhance CD8^+^ T-cell infiltration and intervene in their spatial distribution but also counteracting the cell intrinsic functional impairments that suppress T cells within the TME.

### Suppression of T-cell function within the TME

#### T-cell exhaustion

In response to acute infection, naïve CD8^+^ T cells differentiate into cytolytic effector cells. When acute infections are cleared, memory T cells are generated and poised for long-term protection [213]. However, in the context of chronic infection and cancer, persistent antigen exposure forces antigen-specific CD8^+^ T cells to enter a hyporesponsive state termed exhaustion [111]. In 2007, the Wherry laboratory provided the first systematic definition of CD8⁺ T-cell exhaustion at the molecular level [214]. Through genomic approaches, their study revealed that during chronic viral infection, CD8⁺ T cells enter a distinct differentiation state that is fundamentally different from that of functional effector and memory T cells, establishing a comprehensive transcriptomic signature for this condition and identifying PD-1 as its key surface marker. This seminal work became a cornerstone for understanding T-cell dysfunction in tumor immunology. A subsequent consensus review systematically organized and precisely defined the hallmarks of T-cell exhaustion [215]. Typically, exhausted T cells are characterized by a hierarchical loss of effector functions (e.g., cytokine production and cytotoxic activity) and  proliferative potential, high and sustained expression of inhibitory receptors (e.g., PD-1, CTLA-4, LAG-3, Tim-3, CD39, CD103), and unique transcriptional and epigenetic programs [215, 216]. Moreover, exhausted T cells are enriched in tumor-reactive clones [217, 218]. These features indicate that exhausted T cells are a distinct T-cell lineage that arise during chronic antigen stimulation. It is the main obstacle to adaptive resistance to T-cell-based immunotherapies, including ICI and ICT.

**Fig. 3:**
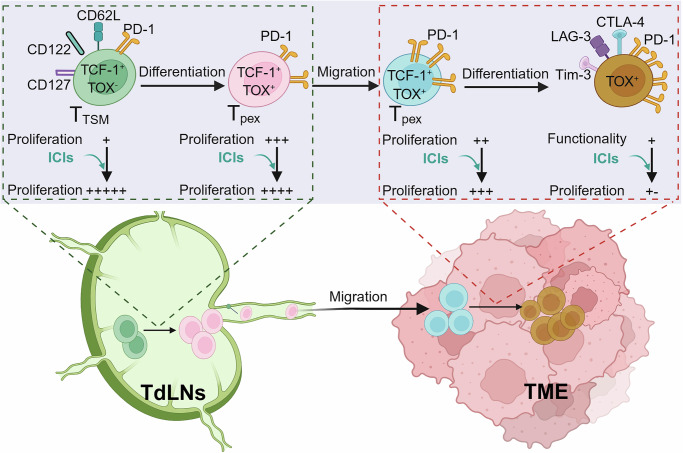
Spatiotemporal differentiation of CD8^+^ T cells from TdLNs to tumors. This schematic illustrates the differentiation trajectory of tumor-specific CD8⁺ T cells in the context of cancer immunotherapy, highlighting the critical difference between memory fate and terminal exhaustion, as well as the key intervention points associated with ICIs. In TdLNs, tumor antigen-presenting DCs prime naïve CD8⁺ T cells, generating activated populations that upregulate the expression of homing and activation markers such as CD62L and PD-1. Among these, antigen-experienced TdLN-T_TSM_ and Tpex subsets—which are characterized by memory-like and stem-like properties (e.g., TCF1⁺)—respond to ICIs and differentiate, with their progeny subsequently infiltrating the tumor bed via the bloodstream. Within the TME, two distinct CD8^+^ T-cell lineages coexist. Tpex cells, which originate from TdLN priming, give rise to terminally exhausted T cells (Tex) under chronic antigen stimulation. Tex cells are characterized by the coexpression of multiple inhibitory receptors (including PD-1, CTLA-4, LAG-3, and TIM-3), upregulation of TOX, and a progressive loss of proliferative capacity and effector function, rendering them largely refractory to ICIs. Therapeutic interventions with ICIs, such as anti-PD-1/PD-L1 antibodies, primarily target the Tpex population

Emerging evidence has revealed that exhausted CD8^+^ T cells constitute a heterogeneous population with specific developmental relationships among their subgroups [219–221]. To improve strategies aimed at reinvigorating exhausted CD8^+^ T cells, including anti-PD therapy, defining the developmental biology of exhausted CD8^+^ T cells and determining which T-cell cluster primarily responds to anti-PD therapy are particularly important. A few years ago, our laboratory defined a unique CXCR5-expressing exhausted CD8^+^ T-cell cluster during chronic infection [222]. This subset exhibits a synergistic effect on controlling viral replication when combined with anti-PD-L1, suggesting its potential to respond to anti-PD treatment. Simultaneously, the Ahmed laboratory has identified a population of virus-specific CD8^+^ T cells that express PD-1 and costimulatory molecules, such as inducible costimulators (ICOSs) and CD28 [223]. This population has a distinct gene signature that is associated with CD4^+^ follicular helper T cells (T_FH_) or CD8^+^ precursor T cells but is distinct from that of terminally differentiated T cells (e.g., T helper 1 or CD8^+^ effectors). This PD-1^+^ CD8^+^ subset exhibits stemness marked  by TCF1 expression and has the ability to respond to anti-PD-1 antibodies, as evidenced  by proliferation and the generation of a new transitional subset with an effector-like transcriptional signature after PD-1 blockade [223, 224]. The PD-1^+^ TCF1^+^ T-cell subset with stemness, also referred to as the Tpex, has subsequently been identified in various tumors, and its pivotal response to PD-1/PD-L1 checkpoint blockade has been confirmed as well [225–227]. Tpex cells reside within the TME, express high levels of TCF1, and are capable of self-renewal, proliferation, and differentiation into terminally exhausted effectors, making them the primary reservoir for sustaining persistent T-cell responses [33]. Unlike short-lived terminally differentiated cells, Tpex cells are a functional cornerstone of multiple immunotherapies. They are not only the core responders to anti-PD therapy but also the superior population in adoptive cell transfer, driving stronger and more durable antitumor effects. Thus, loss of this self-renewing pool impairs T-cell responses and promotes therapeutic resistance. However, PD-1 signaling does not simply exacerbate this loss by suppressing Tpex cells. Rather, it sustains a self‑renewing population of Tpex cells during chronic antigen exposure by precisely balancing their metabolic state and differentiation trajectory [228]. PD‑1 blockade accelerates Tpex differentiation into effector lineages while simultaneously triggering a compensatory feedback mechanism that enhances Tpex self‑renewal to restore pool homeostasis. This dual functionality underscores the complexity of the PD-1 pathway in regulating the balance among exhausted T-cell lineages (Fig. 3). Similarly, TCF1 not only serves as a marker of stem‑like identity but is also essential for optimal T-cell activation and effective anti-PD responses [229]. Improving T-cell priming by manipulating the chromatin accessibility or activity of TCF1 can reverse the defective responses of CD8^+^ T cells upon PD-1 blockade, notably in poorly immunogenic tumors [165, 230]. Interestingly, the Aerts laboratory has also identified CD8^+^ T-cell precursors in TdLNs [231]. These cells can self-renew and respond to PD-L1-directed immunotherapy, with significant prognostic implications. These findings highlight the significant role of some unique tumor-associated CD8^+^ T-cell precursors within different types of tumors in response to PD-1 blockade. More importantly, this influence extends beyond anti-PD therapy, as it critically dictates the durability of the response to CAR-T-cell therapy and emerging checkpoint inhibitors against targets such as LAG-3 and TIGIT. Fortunately, Tpex cells are not fate locked into the exhaustion program; their differentiation trajectory can be modulated by CD4^+^ T-cell help or some factors, such as IL-2 [232–234]. Moreover, a recent study revealed T-bet and CD39 as two critical fate-determining markers that enable the effective stratification of CD8^+^ T cells in TdLNs into two distinct subsets, namely, a T-bet^+^ intermediate-exhausted population associated with ICI therapeutic responsiveness and a CD39^+^ terminally exhausted subset linked to treatment resistance [235]. Accordingly, the expression levels of these two markers in TdLNs can serve as a predictive indicator for ICI therapy outcomes.

These context-dependent effects of PD-1 on Tpex cells are part of the broader role of PD-1 as a major regulator of CD8^+^ T-cell exhaustion. During cancer, continuous antigen stimulation maintains high levels of PD-1 expression on antigen-specific T cells. Prolonged PD-1 expression inhibits the antitumor T-cell response, facilitating tumorigenesis. Thus, the initial therapeutic hypothesis underlying anti-PD therapy involves restoring the exhaustion of PD-1^+^ CD8^+^ T cells by releasing their “brakes”. Indeed, the effects of blocking the PD-1 pathway on the restoration of T-cell function have been shown in many preclinical and clinical studies. However, persistent loss of PD-1 signaling has both beneficial and detrimental effects on CD8^+^ T-cell function and fate. In contrast to wild-type cells, CD8^+^ T cells with PD-1 deficiency proliferate earlier during chronic infection but become hypofunctional and more prone to death at later stages of infection [236]. On the one hand, this implies that T-cell exhaustion most likely limits the durable anti-PD response, acting as a critical pathway of resistance to anti-PD therapy. On the other hand, although the primary goal of anti-PD therapy is to address T-cell exhaustion, when T-cell exhaustion is taken to extreme limits, anti-PD treatment may paradoxically promote the development of anti-PD resistance. Moreover, PD-1 expression on exhausted CD8^+^ T cells is heterogeneous, and heterogeneous PD-1 expression likely affects sensitivity to anti-PD-1 therapy [237]. As such, both PD-1^low^ and PD-1^intermediate^ exhausted T cells retain the capacity to be reinvigorated by anti-PD-1 therapy, while the PD-1^high^ terminally exhausted T-cell subset is closely associated with anti-PD resistance. A study of chronic infection from the Wherry laboratory revealed that PD-1 ^intermediate^  CD44^high^ exhausted CD8^+^ subset could be reinvigorated by PD-L1 blockade, whereas the PD-1^-high^ CD44^-intermediate^ subset appeared more terminally differentiated and responded poorly to PD-L1 blockade (Fig. 3). Therefore, a durable response to anti-PD therapy likely depends on a certain level of PD-1 expression on exhausted T cells with differentiation capacity. In addition to expressing PD-1, exhausted T cells coexpress a repertoire of other inhibitory receptors, including LAG-3, Tim-3, and TIGIT. These receptors constitute a synergistic checkpoint network, where targeting PD-1 alone often fails because of the compensatory upregulation of these alternative pathways. Thus, therapeutic strategies designed to improve the recovery from exhaustion, such as combination checkpoint blockade (e.g., anti-PD-1 plus anti-LAG-3) or the development of novel bispecific CAR-T cells targeting PD-L1 and other coexpressed receptors, are fundamentally based on this principle [238–240].

**Fig. 4:**
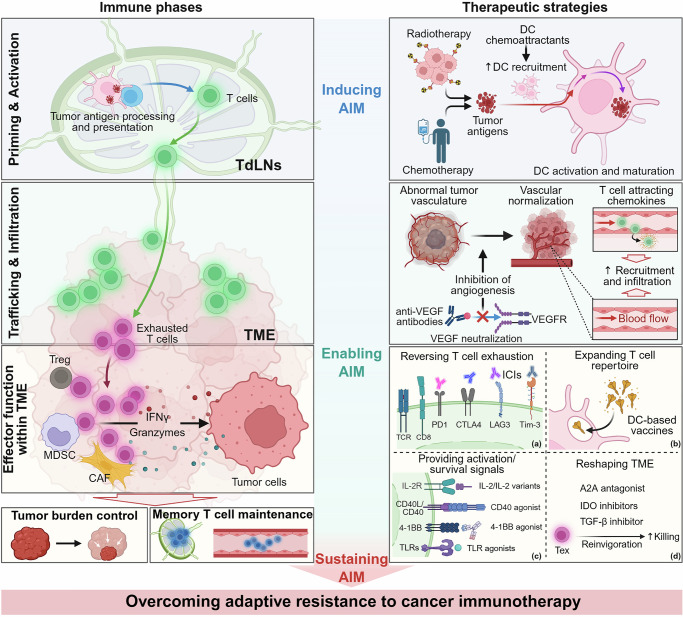
Roadmap to overcome adaptive resistance in cancer immunotherapy. The schematic organizes therapeutic strategies according to the three sequential phases of antitumor immunity required for establishing durable AIM. The left panel depicts immune phases related to antitumor immunity, comprising three key phases arranged from top to bottom: (1) priming and activation, which involves tumor antigen processing and presentation from DCs and initial T-cell activation; (2) trafficking and infiltration, which encompasses T-cell migration from TdLNs into the TME; and (3) effector function within the TME, where T cells exert cytotoxicity, navigate metabolic competition, and are subject to immune checkpoint regulation and exhaustion. The middle panel outlines strategic goals corresponding to each immune phase; the aims are defined as inducing AIM (initiating antitumor immunity), enabling AIM (facilitating T-cell entry), and sustaining AIM (maintaining T-cell fitness and promoting memory differentiation). The right panel shows therapeutic strategies aligned with their respective immune phases. To sustain the AIM, interventions are further categorized into four complementary mechanisms that reflect the multilayered approach required for durable responses: (**a**) reversing T-cell exhaustion: ICIs targeting PD-1, CTLA-4, LAG-3, and Tim-3 restore effector function in exhausted T cells; (**b**) expanding the T-cell repertoire: DC-based vaccines broaden the pool of tumor-specific T cells; (**c**) providing activation/survival signals, such as engineered IL-2 variants and costimulatory agonists (e.g., 4-1BB and CD40), and TLR agonists enhance T-cell activation, proliferation, and functional persistence; (**d**) reshaping the TME: inhibitors of VEGF, TGF-β, IDO, and adenosine A2A receptor remodel the immunosuppressive microenvironment to support T-cell infiltration, function, and longevity; and (**e**) promoting memory differentiation and maintenance to achieve durable responses. Collectively, this framework illustrates how rationally designed combination strategies targeting distinct immunological bottlenecks can synergize to achieve durable responses and overcome adaptive resistance

Beyond the PD-1-centered regulatory network, recent studies have revealed that Tpex cells exhibit significant temporal heterogeneity over the course of chronic antigen exposure. In both acute and chronic infection models, Tpex cells can be detected as early as the priming phase, emerging alongside effector T-cell differentiation [241, 242]. Notably, early-stage Tpex cells display robust proliferative capacity and maintain a transcriptional landscape reminiscent of that of memory T cells (T_mem_). However, as chronic infection progresses to the middle and late stages, Tpex cells undergo progressive functional decline, characterized by markedly reduced proliferative potential and a gradual shift toward terminal exhaustion. This temporal deterioration underscores that Tpex cells are not a static population but rather a dynamic entity whose functional properties evolve over time. Emerging evidence also suggests that Tpex cells may interconvert with T_mem_ cells under certain conditions, challenging the view that exhaustion represents an irreversible differentiation trajectory [243]. This plasticity has important implications for cancer immunotherapy, as it raises the possibility of redirecting Tpex cells toward a memory fate rather than simply reinvigorating them within the exhaustion program. In parallel with these observations on Tpex plasticity, our laboratory recently identified another distinct memory CD8^+^ T-cell in TdLNs, termed TdLN-T_TSM_ [167]. TdLN-T_TSM_ cells are characterized by a TCF1^+^ TOX^-^ PD-1^low^ phenotype and exhibit canonical memory features, including high expression of memory-associated markers (e.g., CD127, CD122, and CD62L), as well as transcriptomic and epigenomic profiles resembling those of conventional T_mem_ cells. Notably, in contrast to previously reported Tpex cells in either TdLNs or the TME, TdLN-T_TSM_ cells show superior proliferative and antitumor capacity without carrying an epigenetic scar. De novo transfer of TdLN-T_TSM_ cells rescues lymphadenectomy-induced resistance to anti-PD-L1 therapy when administered before or during treatment, confirming that TdLN-T_TSM_ cells are bona fide responders to PD-L1 blockade. Given the early emergence of Tpex cells and their plasticity toward memory, an important question arises regarding the relationship between early Tpex cells and the TdLN-T_TSM_ subset. We propose that TdLN-T_TSM_ cells may represent an early-stage or precursor Tpex population that retains full memory potential, whereas Tpex cells that have resided in the TME for extended periods become progressively restricted to the exhaustion trajectory. This distinction highlights whether Tpex cell generation occurs in TdLNs during the priming phase or within the TME under chronic stimulation critically influences their functional properties and therapeutic responsiveness. These temporal and spatial considerations have direct implications for overcoming adaptive resistance. Early-stage Tpex cells, with their preserved memory-like features and high proliferative capacity, may be more amenable to reinvigoration by immunotherapy. These cells represent the primary responders to ICI therapy, and strategies aimed at expanding this pool, such as stimulation with IL-2 or IL-15, could further enhance therapeutic efficacy [244, 245]. In contrast, late-stage Tpex cells, which have undergone functional decline, may require alternative approaches, such as combination therapies targeting metabolic pathways or epigenetic modifiers, to restore their functionality. Examples include modulating the AMP-activated protein kinase (AMPK) or mTOR pathways to improve mitochondrial fitness or using HDAC inhibitors or DNA methyltransferase inhibitors to reprogram these cells toward a more functional state [246–248]. More broadly, these stage-specific strategies align with the framework outlined in Fig. 4, where “Inducing AIM” focuses on priming early T-cell responses, “Enabling AIM” facilitates the infiltration and function of early-stage effectors, and “Sustaining AIM” seeks to maintain long-term memory populations. By tailoring interventions to the temporal state of Tpex cells, it may be possible to achieve more durable AMIs and overcome adaptive resistance. Critically, the induction of T cells with a memory phenotype under conditions of persistent antigen exposure represents a key mechanism underlying the durable efficacy of anti-PD therapy. Therefore, in-depth investigations into how to promote T-cell memory formation and how to leverage these memory T cells therapeutically could substantially increase immunotherapy sensitivity and facilitate the establishment of durable AIM.

On the other hand, functionally exhausted CD8^+^ T cells exhibit antigen addiction, and persistent antigen impedes the formation of memory subsets in tumor or chronic infection models [249]. Nonetheless, the formation of T cells with the capacity to provide long-term protective immunity is not precluded. A study by the Ribas laboratory revealed that tumors from individuals who developed anti-PD-1 resistance had significantly fewer effector memory CD8^+^ T cells than those from patients who responded positively to treatment [250]. This memory T-cell subset is predominantly expanded following PD-1 blockade and is largely restricted by anti-PD-1 resistance. In addition, the Wilmott laboratory has defined an EOMES^+^ CD69^+^ CD45RO^+^ effector memory T-cell phenotype within tumors that is more abundant in responders to the combination of anti-PD-1 therapy and anti-CTLA-4 therapy than in nonresponders [251]. Their findings further support the conclusions drawn by Ribas. Thus, overcoming T-cell exhaustion-associated therapeutic resistance requires a multipronged strategy focusing on several directions. First, targeting the epigenetic landscape could reprogram terminally exhausted T cells into a more functional state. Second, rational combination therapies that concurrently block the synergistic inhibitory network are essential. Furthermore, T cells can be engineered by editing exhaustion- or memory-related genes. Finally, modulating the cytokine milieu can steer T cells toward durable memory-like or Tpex-like states. Collectively, these approaches aim to fundamentally alter the fate and function of T cells for the sustained efficacy of cancer immunotherapy.

#### Coexpression of other inhibitory checkpoint molecules in addition to PD-1/CTLA-4

In addition to PD-1, T cells with an exhausted phenotype are characterized by the coexpression and activation of a repertoire of other inhibitory receptors, including Tim-3, CLTA-4, LAG-3, NKG2A, and TIGIT, which collectively constitute a synergistic functional inhibitory network. This phenomenon underpins a central mechanism of treatment resistance: anti-PD monotherapy may confer compensatory upregulation of other checkpoint molecules (e.g., LAG-3 and Tim-3) on T cells, thereby amplifying localized T-cell suppression [252, 253]. Thus, the upregulation of these alternative immune inhibitory checkpoints is closely associated with both adaptive anti-PD resistance and the restrictive durability of ICT therapies [253]. These findings not only highlight the necessity for combinational strategies but also provide a theoretical foundation for understanding adaptive resistance to cancer immunotherapy and for designing rational combination regimens. The drivers of this checkpoint network are multifaceted. While TCR activation and its downstream pathways are primary initiators of checkpoint expression (a process distinct from the state of exhaustion itself), a complex cytokine network shaped by the TME also plays a critical role. Additional TCR-independent mechanisms, particularly those involving cytokines, act cooperatively to promote the expression of these immune checkpoints. The common γ-chain cytokines IL-2, interleukin-7 (IL-7), IL-15, and interleukin-21 (IL-21) increase PD-1 and Tim-3 expression on T cells [254, 255]. IL-12 and interleukin-27 (IL-27) from the IL-12 family of cytokines are crucial for Tim-3 expression [256, 257]. IL-27 even widely influences TIGIT, LAG-3, CTLA-4, and PD-1 expression by activating STAT1 and B lymphocyte-induced maturation protein 1 (BLIMP1) [258]. Other cytokines, such as IL-6, IL-10, TGF-β, and IFNs, are also positively associated with checkpoint expression [259–263]. Similar observations have been reported to be induced by noncytokine factors, including hypoxia-inducible factor-1 alpha (HIF-1α), epigenetic and transcriptional regulation or posttranscriptional and posttranslational regulation of checkpoints [264–267]. For example, key coinhibitory receptors such as PD-1, LAG-3, and TIGIT are coordinately upregulated in T cells through a shared transcriptional regulatory module involving c-Fos and JunB [258]. This coordinated expression accounts for their frequent coexpression in functionally exhausted T cells and reveals the underlying mechanism by which these receptors are regulated as an integrated functional unit. Importantly, these immune checkpoints synergistically rather than redundantly drive T-cell exhaustion. Emerging evidence indicates that LAG‑3 primarily disrupts TCR signal transduction, whereas PD‑1 attenuates costimulatory signals [263]. The combined inhibition mediated by these receptors leads to a more profound suppression of T-cell functions, particularly IFN-γ secretion, which is required for antitumor immunity. These insights provide a mechanistic foundation and strategic framework for the development of LAG-3/PD-1 bispecific antibodies and rational combination immunotherapies.

Although a combinatorial blockade strategy targeting PD-1 plus LAG-3 or PD-1 plus Tim-3 has been demonstrated to improve the anti-PD response, some issues remain unclear. First, these studies highlight that different PD1^+^ T-cell subsets play distinct antitumor roles. Therefore, future research should focus on determining how to specifically target these T-cell subsets to overcome acquired anti-PD resistance. Second, a preclinical study revealed that triple blockade targeting PD-1, LAG-3, and CTLA-4 resulted in 20% tumor-free survival (TFS), whereas dual inhibition of LAG-3 and CTLA-4 in PD-1 null mice resulted in a TFS of 40%, indicating a hierarchical ordering of checkpoint functions. Third, the dominant immunological checkpoints and the variations in their activity across different tumor types or stages of tumor development have yet to be fully elucidated. Next, the expression of Tim-3, LAG-3, and V-domain immunoglobulin suppressor of T-cell activation (VISTA) increases when anti-PD resistance is achieved; however, whether these alterations are directly linked to resistance is unclear. Finally, the side effects caused by drug interactions are also potential risks that should be considered when drugs targeting other checkpoints in combination with anti-PD therapy are used. Considering that the upregulation of these checkpoints may occasionally be linked to T-cell exhaustion or depletion [268, 269], the mechanisms that promote the induction of T-cell exhaustion are not completely clear. Even with many of the studies mentioned above, confirming or identifying the specific mechanisms involved in ICI resistance can also be challenging.

 Furthermore, while dual or triple blockade of PD-1 and other aforementioned factors can, to some extent, enhance T-cell cytotoxicity capacity and improve the efficacy of ICI monotherapy, it remains unclear whether this functional restoration results directly from the inhibition of specific inhibitory receptors or indirectly from broader modulation of the tumor immune microenvironment. Moreover, it is still not completely clear whether reducing PD-1 expression among exhausted PD-1^high^ CD8^+^ T cells enhances sensitivity to anti-PD therapy or whether the expression of checkpoints, such as LAG-3, Tim-3, B and T lymphocyte attenuator (BTLA), and VISTA, can be independent of PD-1. Further characterization of exhausted T cells in the context of anti-PD resistance is therefore needed. Additionally, the relative contributions of each targeted pathway and whether the observed improvements in T-cell function translate to durable anti-PD responses across different types of cancer treated with different combination regimens need to be determined. Future therapeutic strategies should pivot from mere receptor blockade to targeting the core upstream drivers of checkpoint expression, such as epigenetic reprogramming, key cytokine signaling, or metabolic dysregulation, to fundamentally reverse the T-cell exhaustion program. Finally, the diverse immune profiles and tumor heterogeneity across patients may influence the efficacy of anti-PD treatment, highlighting the need for the development of personalized approaches and biomarkers to overcome anti-PD resistance and guide more precise combinational therapeutic decisions to adjuvant PD-1/PD-L1 blockade therapy.

#### Context-dependent roles of IFN signaling in both stimulatory and suppressive antitumor immunity

IFNs, comprising type I (IFN-α/β) and type II (IFN-γ), are pivotal cytokines that play dual roles in antitumor immunity [270, 271]. As central mediators bridging innate and adaptive immunity, IFNs activate JAK-dependent signaling pathways and serve as critical components of immune surveillance against cancer. On the one hand, they exert potent antitumor effects by directly inhibiting tumor proliferation, enhancing antigen presentation, and activating effector T cells. On the other hand, persistent IFN signaling has been shown to drive T-cell exhaustion, foster an immunosuppressive tumor microenvironment, and even promote tumor immune evasion. This functional duality underscores the IFN pathway as a central hub in understanding and improving cancer immunotherapy.

IFNs orchestrate effective antitumor immune responses through multidimensional and multicellular synergistic mechanisms. Type I IFNs serve as critical bridges connecting innate and adaptive immunity. They not only directly inhibit tumor cell proliferation and induce apoptosis but also, more importantly, potently activate DCs, promoting their maturation and antigen-presenting function, thereby laying the foundation for cytotoxic T-cell activation [272–275]. A recent study further demonstrated that type I IFN can induce a specific subset of CD11b-positive conventional dendritic cells (cDC2s) to take up and express tumor-derived MHC-I molecules, thereby efficiently cross-presenting tumor antigens to CD8^+^ T cells [276]. On the other hand, clinical observations have revealed that elevated peripheral type Ⅰ IFN levels prior to ICI treatment are associated with longer PFS in NSCLC patients [277]. Similarly, enhanced tumor-derived IFN-α/β production strongly inhibits tumor growth and has synergistic effects with ICI therapy. Moreover, the engineering of ICIs (e.g., anti-PD antibodies) conjugated with IFN-α or PEGylated IFN-α creates a feedforward response that overcomes resistance to both type I IFNs and ICIs with minimal side effects in advanced tumors [278–281]. Therefore, across various preclinical models and early-stage clinical trials, type I IFN-based therapies (including recombinant IFN-α and IFN gene agonists) have been shown to enhance tumor immunogenicity and synergistic effects with ICIs, establishing type I IFN responses as favorable prognostic biomarkers for ICI efficacy [282]. Similarly, novel immunotherapies utilizing recombinant type I IFNs, IFN-encoding vectors, or IFN-expressing cells are under active development. Moreover, the negative effects of type I IFNs on antitumor immunity, including the induction of immunosuppressive molecules, the enhancement of Treg function, and the promotion of T-cell exhaustion, should also be recognized [283]. Complementing type I IFNs, type II interferon (IFN-γ), which is produced primarily by activated T cells and NK cells, acts as a central executor of effector immune responses. Through its role in the classical JAK-STAT signaling pathway, IFN-γ upregulates the expression of both MHC-I and MHC-II molecules on tumor cells, increasing their susceptibility to T-cell-mediated recognition and elimination. Moreover, it enhances antigen presentation machinery in DCs and other APCs, promotes T-cell recruitment to tumor sites, and activates the JAK/STAT/IRF1 signaling pathway to induce direct cytotoxic effects on cancer cells [284–287]. Furthermore, it induces the polarization of macrophages toward the antitumor type 1 macrophage (M1) phenotype and suppresses tumor angiogenesis [288]. Consequently, IFN-γ and its associated gene signatures are frequently utilized as potential biomarkers for predicting clinical responses to ICIs and other immunotherapies for various tumors [289].

However, the opposite side of IFN’s “double-edged sword” nature becomes particularly evident in clinical treatment resistance. Persistent exposure to IFN signaling, especially IFN-γ, strongly selectively drives tumor cells to evade its cytotoxic effects through genetic mutations or epigenetic alterations in IFN signaling pathways following ICI therapy [290]. In brief, adaptive therapeutic resistance can develop because of mutations that render tumor cells insensitive to IFN-γ. The most classic mutation is a loss-of-function mutation in components of the JAK-STAT signaling pathway. In patients who develop resistance following cancer immunotherapy (e.g., anti-PD-1/PD-L1 antibodies), their tumor cells frequently harbor truncating mutations in JAK1 or JAK2. These mutations cause tumor cells to numb to IFN-γ-mediated growth inhibition and apoptotic signals while simultaneously impairing their ability to upregulate the expression of PD-L1 and MHC molecules [291]. A recent study confirmed that among 1201 patients with NSCLC, more than 60% of initial responders to PD-L1 blockade relapse, and adaptive resistance to PD-L1 blockade in NSCLC is associated with ongoing but altered inflammation and IFN signaling [83]. Relapsed tumors can be separated by upregulated or stable expression of IFN-γ response genes, which is associated with putative routes of resistance characterized by signatures of a persistent inflamed TME, immune dysfunction, and mutations in antigen-presenting genes. In addition to the classical IFN-γ/JAK/STAT axis, persistent type I IFN signaling has been linked to therapeutic resistance. It can shape a profoundly immunosuppressive TME by inducing the expansion of myeloid cells, enhancing the suppressive function of Tregs, or directly driving CD8^+^ T cells toward terminal exhaustion, thereby undermining the efficacy of various immunotherapies [292–296]. This dual evasion strategy enables tumors to neither be killed nor recognized.

Specifically, persistent IFN signaling sustains progressive CD8^+^ T-cell differentiation toward a terminal-exhausted state, which is associated with the restriction of antitumor immunity and the failure of immune treatment [96, 296]. In some cases, IFN-related mutations or signaling pathway alterations can lead to antigen presentation defects. For example, mutations in the JAK pathway can disrupt the normal antigen presentation process and contribute to resistance to anti-PD treatment [297]. A recent study indicated that IFN-α rewired glucose metabolic cross-talk within the TME of hepatocellular carcinoma, thereby liberating CD8^+^ T-cell cytotoxic capacities and potentiating the PD-1 blockade-induced immune response [298]. Other immune cells within the TME can also be influenced by IFN signaling. IFNs polarize macrophages into either the M1 phenotype, which has antitumor activity, or the type 2 macrophage (M2) phenotype, which can promote tumor growth. For example, in the context of anti-PD treatment, the balance between these macrophage phenotypes is important. Although IFN-γ suppresses tumor-derived chemokines to inhibit the tumor trafficking of CXCR2^+^CD68^+^ macrophages (associated with M2 polarization) by blocking the CXCL8-CXCR2 axis, thereby counteracting immunosuppression and overcoming therapeutic resistance, dysregulated IFN signaling can promote M2-skewed macrophage polarization, which compromises the efficacy of anti-PD-1/PD-L1 therapies [299]. Disruption of this balance directly affects the ultimate efficacy of various therapies, such as adoptive cell therapy, cancer vaccines, and oncolytic viruses. Disruption of this balance also significantly impairs the therapeutic outcomes of various immunotherapeutic approaches, such as cancer vaccines and oncolytic virus treatments [300, 301].

Indeed, blocking type I IFNs has been shown to improve antiviral and antitumor immune responses in both chronic lymphocytic choriomeningitis virus infection and tumors [279, 302, 303]. However, ongoing studies have revealed that the multifaceted role of IFNs in the antitumor response during anti-PD treatment involves not only the classical JAK-STAT signaling pathway but also various noncanonical pathways that can lead to diverse outcomes, contributing to either tumor progression or tumor regression under certain conditions. Therefore, confronted with the dual nature of IFN signaling, future cancer immunotherapy strategies must embrace greater precision and dynamic regulatory capabilities. One promising strategy involves the development of combination therapies that integrate established immunotherapies (such as ICIs and CAR-T cells) with pharmaceutical agents capable of modulating IFN signaling. For instance, when low-dose IFN-α is used during the early stages of treatment, it may serve as an immune primer to enhance initial antitumor responses, whereas the timely introduction of small-molecule inhibitors targeting IFN signaling or JAK inhibitors upon signs of T-cell exhaustion could help restore T-cell functionality. Additionally, cotargeting alternative immune checkpoints upregulated by IFN signaling (e.g., LAG-3 and TIGIT) represents a rational and promising therapeutic avenue. In cases of resistant tumors harboring JAK mutations that impair IFN signal transduction, novel immunotherapeutic approaches that bypass this defective pathway (such as those leveraging IFN-independent cytotoxic mechanisms) or bispecific antibodies offer a critical strategy to overcome treatment resistance. Furthermore, the integration of genomic and transcriptomic profiling to develop refined IFN-related gene signatures holds significant potential for pretreatment patient stratification, enabling the identification of individuals who are likely to benefit from enhanced IFN signaling, as well as those whose tumor microenvironments may already be characterized by chronic IFN exposure and associated immunosuppressive effects. Such insights would facilitate personalized therapeutic decision-making. In summary, the IFN signaling network plays a dual role in cancer immunotherapy, acting both as an enhancer of antitumor immunity and as a contributor to immune dysfunction. Breakthroughs in future therapies will largely depend on our ability to more precisely dissect and strategically manipulate this central pathway, ultimately directing powerful offensive capabilities of the immune system against tumors with improved persistence, specificity, and efficacy.

#### T-cell death as a barrier to sustained antitumor immunity

Beyond functional exhaustion and the complex signaling networks that regulate T-cell activity, the physical elimination of tumor-reactive T cells within the TME represents a critical—yet often overlooked—mechanism that limits the durability of antitumor immunity. Multiple forms of T-cell death, including apoptosis, ferroptosis, and necroptosis, contribute to the loss of tumor-specific T cells and promote adaptive resistance to immunotherapy. Apoptosis is a central mechanism of T-cell death within the TME. Chronic antigen stimulation and persistent TCR signaling upregulate death receptors such as Fas (CD95) and tumor necrosis factor (TNF)-related apoptosis-inducing ligand (TRAIL) receptors on activated T cells, increasing their susceptibility to activation-induced cell death (AICD) [304]. Moreover, immunosuppressive cells within the TME, including Tregs and MDSCs, can induce T-cell apoptosis through Fas ligand expression or the secretion of proapoptotic cytokines such as TNF-α and TGF-β [305]. The balance between pro-apoptotic and anti-apoptotic signals, which is regulated by the Bcl-2 family of proteins, critically determines T-cell persistence [306]. Accordingly, strategies that increase the expression of antiapoptotic proteins (e.g., Bcl-2 and Bcl-xL) in adoptively transferred T cells have been shown to improve their survival and antitumor efficacy. Ferroptosis, an iron-dependent form of regulated cell death driven by lipid peroxidation, has emerged as a significant barrier to T-cell persistence in the TME. Tumor-associated metabolic stress, characterized by glutathione depletion, iron overload, and reactive oxygen species (ROS), creates an environment permissive to ferroptosis [307]. Notably, CD8⁺ T cells within the TME exhibit increased susceptibility to ferroptosis because of lipid peroxidation and the downregulation of the activity of antioxidants such as glutathione peroxidase 4 [308]. This process is further exacerbated by nutrient competition with tumor cells, which depletes cysteine and glutamine—two critical amino acids required for glutathione synthesis. Importantly, emerging evidence suggests that resistance to immunotherapy may be partially attributed to ferroptosis-mediated loss of tumor-reactive T cells, and pharmacological inhibition of ferroptosis has been shown to increase T-cell persistence and improve responses to immunotherapy [309]. Other forms of T-cell death, including necroptosis and pyroptosis, may also contribute to T-cell attrition within the TME, although their roles in the context of cancer immunotherapy remain less well characterized [310, 311].

Collectively, these cell death pathways represent a critical bottleneck for sustained antitumor immunity. Strategies aimed at preventing T-cell death hold promise for enhancing the durability of immunotherapy. These include engineering T cells with enhanced expression of antiapoptotic factors such as Bcl-2 or Bcl-xL to confer resistance to AICD, pharmacological inhibition of ferroptosis using small molecules such as ferrostatin-1 or liproxstatin-1 to preserve T-cell viability under metabolic stress, and combination approaches that simultaneously target cell death pathways and immune checkpoint blockade to prolong T-cell survival and sustain antitumor responses [312]. These approaches align closely with the “sustaining AIM” pillar of our strategic framework (Fig. 4), as preserving T-cell viability is essential for establishing durable AIM. Addressing T-cell death as a barrier to durable immunity thus represents an emerging frontier in overcoming adaptive resistance to cancer immunotherapy.

## Emerging therapeutic strategies to overcome adaptive resistance

Although cancer immunotherapy has shown an improved safety profile over conventional treatments such as chemotherapy and radiotherapy, its clinical success remains limited by the high incidence of adaptive resistance [313]. Many initial responders eventually experience relapse, a process driven by tumor immunoediting under therapeutic pressure, which disrupts the induction and maintenance of AIM. Through targeting mechanisms such as antigen loss, T-cell exhaustion, and immunosuppressive TME remodeling, tumors not only achieve immune evasion but also critically impair the formation of durable memory T-cell responses, which are essential for long-term protection. Therefore, immunotherapies require shifting the focus from enhancing tumor killing to guiding the immune system toward establishing sustained AIM. To this end, it is essential to develop a comprehensive therapeutic framework that addresses the distinct phases of the antitumor immune response. First, therapies that can counteract early immunoediting and establish a robust T-cell response are prerequisites for the development of AIM. Second, strategies that enable AIM should focus on reversing T-cell exhaustion and guiding T-cell fate, thereby ensuring that activated T cells differentiate into functional effectors rather than becoming exhausted. Finally, to achieve long-term protection, interventions aimed at sustaining AIM must emphasize the development of long-lived memory cells and prevent the recurrence of exhaustion. This section explores how these approaches enhance the efficacy of AIM and thereby accelerate progress toward a functional cure. A summary of the discussed strategies is presented in Fig. 4.

### Combination therapies: from reversing exhaustion to inducing lasting AIM

Rationally designed combination therapy is key for overcoming immune editing and inducing durable antitumor immunity. Among them, the combination of ICIs with radiotherapy or chemotherapy constitutes the basic strategy. This strategy can induce immunogenic cell death and enhance antigen presentation, thereby priming the TME for enhanced ICI efficacy and establishing the necessary inflammatory context for the subsequent formation of AIM [314–316]. Clinically, this synergistic strategy has demonstrated substantial survival benefits across tumors. In cancers such as NSCLC and TNBC, this combination has become a cornerstone of first-line therapy [317–321]. Beyond these, its utility extends to other aggressive cancers, including small cell lung cancer (SCLC), esophageal squamous cell carcinoma, and high-grade digestive neuroendocrine neoplasms, where it has consistently improved outcomes and is supported by clinical guidelines [322–325]. Collectively, these trials validate that disrupting the immunosuppressive landscape through conventional therapies can effectively set the stage for durable immune responses, a possible prerequisite for AIM establishment.

The biological foundation for combination strategies centers on reversing T-cell exhaustion, a key barrier to establishing durable antitumor immunity. Research has shown that PD-1/PD-L1 blockade primarily rescues less exhausted CD8^+^ T cells expressing intermediate levels of PD-1, whereas terminally exhausted cells with high PD-1 expression respond poorly [326]. This evidence highlights that a preexisting reservoir of antigen-specific T cells with memory or precursor potential is crucial for initiating an effective response to ICIs. Therefore, rational combination therapies should aim not only to rescue exhausted cells but also to actively expand these functional T-cell populations and guide their differentiation toward long-lived memory states, ultimately prolonging the response to immunotherapy. Following this rationale, combining immunotherapy with agents that enhance T-cell priming, expansion, and survival can generate critical synergy. A prime example is the proinflammatory cytokine IL-2, which potently promotes T-cell activation and proliferation. Preclinical and clinical evidence indicates that ICIs combined with IL-2 substantially trigger an alternative differentiation pathway in CD8⁺ precursor cells, leading to a transcriptionally and epigenetically distinct effector population with enhanced functionality [234]. Importantly, next-generation IL-2 variants, such as engineered IL-2Rβγ-biased agonists or bispecific PD‑1‑targeted interleukin‑2 variant molecules that avoid CD25 binding, are designed to preferentially expand memory-like or stem-like tumor-reactive CD8⁺ T cells while modulating the exhaustion program and reprogramming the immunosuppressive TME [234, 244, 327–331]. These approaches not only break adaptive resistance but also provide a lasting cellular reservoir essential for the formation of sustained AIM.

Additionally, dual-checkpoint inhibition has shown promise in overcoming adaptive resistance to immune monotherapy. The CheckMate-012 trial provides the first clinical evidence of superior antitumor efficacy with anti-PD-1 plus anti-CTLA-4 in NSCLC patients, thereby supporting further evaluation of this approach in a phase 3 trial [332]. The most recent publication indicates that PD-1/CTLA-4 dual strategies are needed. Building on the foundation of PD-1/CTLA-4 inhibition, targeting the next generation of immune checkpoints, including LAG-3, TIGIT, and Tim-3, represents a pivotal strategy to counteract adaptive resistance by more comprehensively reversing T-cell exhaustion [333–337]. For instance, LAG-3 sustains the expression of the exhaustion-associated transcription factor TOX, and its blockade, particularly when combined with PD-1 inhibition, can increase CD8⁺ T-cell cytotoxicity [263, 338]. Critically, however, emerging evidence suggests that modulating these pathways differentially impacts T-cell fate. While LAG-3 blockade potently enhances effector function, it may do so at the expense of long-term T-cell persistence, potentially constraining the establishment of durable AIM [238]. This highlights the nuanced, nonredundant roles of these checkpoints in regulating T-cell exhaustion, suggesting the possibility of better addressing anti-PD adaptive resistance in a way that relies on restoring T-cell function. Clinically, dual-checkpoint inhibition has validated this mechanistic synergy. Compared with nivolumab plus ipilimumab, the combination of nivolumab and relatlimab (anti-LAG-3) improved PFS and OS in advanced melanoma patients, with a superior safety profile [339, 340]. More importantly, its durable clinical activity in patients who have progressed on prior anti-PD-1/PD-L1 or CAR-T-cell therapy underscores its potential to overcome adaptive resistance [341]. Similarly, tiragolumab (anti-TIGIT), which can remodel the immunosuppressive TME and favor a memory-like CD8^+^ T-cell phenotype, has shown enhanced efficacy when combined with atezolizumab [342]. Targeting Tim-3, a checkpoint upregulated in PD-1-resistant patients, also holds promise for restoring T-cell function and reversing adaptive resistance [343]. Encouraging results extend to the neoadjuvant setting, where a PD-1/LAG-3 bispecific antibody combined with other ICIs resulted in superior outcomes in patients with resectable melanoma [344]. Above all, next-generation checkpoint inhibitors provide essential tools for reversing the multilayered T-cell exhaustion induced by immunoediting. The key future challenge lies in rationally combining these agents—not only to reinvigorate effector responses but also, more critically, to selectively support the survival and expansion of memory precursor T cells. This dual approach would foster a more robust and durable AIM needed for long-term disease control.

To complement direct checkpoint blockade, an equally fundamental strategy for counteracting adaptive resistance involves broadening the repertoire and increasing the frequency of tumor-specific T cells using DC-based vaccines. By harnessing the most potent professional APCs loaded with tumor-associated or tumor-specific antigens, this approach primes naïve T cells either ex vivo or in situ, thereby expanding the tumor-reactive T-cell pool and establishing a broader immunological baseline for subsequent modulation [345]. The FDA-approved PROVENGE (sipuleucel-T) for metastatic castration-resistant prostate cancer exemplifies the clinical feasibility of this strategy [346]. Importantly, while direct checkpoint blockade aims to reverse T-cell exhaustion, DC-based vaccines address the upstream limitation of insufficient tumor-specific T-cell precursors. This complementary mechanism synergizes with checkpoint inhibitors and immune agonists to simultaneously increase both the quantity of tumor-specific T cells and the quality of their functional persistence, ultimately resulting in a more robust and durable AIM.

Once the tumor-specific T-cell repertoire has been expanded, a second layer of modulation involves providing potent activation and/or survival signals using immune-modulating biologic agents. These agents include agonists targeting costimulatory receptors such as CD137 (e.g., urelumab or anti-4-1BB/PD-L1 bispecific antibody) [347–350], CD20 (e.g., rituximab) [351, 352], CD27 (e.g., varlilumab, augments T-cell proliferation) [353, 354], OX40/OX40 ligand [355], CD80 [356], as well as agents like CD40 agonists (e.g., sotigalimab) that enhance antigen presentation by activating DCs and macrophages, which improves antigen presentation and primes a more robust T-cell response [357, 358]. Key insight for AIM-directed therapy comes from the ICOS pathway. In contrast to typical costimulation, sustained ICOS signaling during chronic antigen exposure can impair the memory-like properties of exhausted CD8⁺ T cells. Conversely, blocking the ICOS ligand preserves and enhances the memory-like phenotype of exhausted precursor T cells, thereby improving tumor control and synergizing with PD-1/PD-L1 blockade [359]. These findings highlight the critical principle that the role of costimulatory pathways is context dependent and that their therapeutic manipulation must be precisely tailored to favor the generation of durable AIM, not just transient activation. Other immune modulators, such as Toll-like receptor (TLR) agonists (e.g., vidutolimod, imiquimod, or poly (I:C)) [360, 361] and cytokines such as IL-6 [362, 363], can further reshape the TME to foster a more immunogenic and T-cell-supportive milieu. Beyond directly modulating T cells, reshaping the immunosuppressive TME is equally critical for establishing a permissive niche for AIM. Inhibiting key suppressive factors, such as VEGF, TGF-β, and adenosine, can synergize with immunotherapy by improving T-cell infiltration, function, and longevity. For instance, the anti-VEGF antibody bevacizumab not only normalizes tumor vasculature but also counteracts inhibitory cytokines such as IL-10, thereby enhancing immunotherapy efficacy [364]. Similarly, coblockade of TGF-β and PD-1 has been shown to remodel the T-cell-deficient TME and restore T-cell activity, offering a promising strategy even in challenging settings such as those associated with EGFR-mutated NSCLC [187]. Pharmacological inhibition of the adenosine A2A receptor can also reverse myeloid-mediated immunosuppression and rescue CD8⁺ T-cell function, sensitizing tumors to similar metastatic castration-resistant prostate cancer to atezolizumab and inducing a favorable ORR [182]. In summary, the use of immune agonists and TME-modulating agents not only amplifies immediate effector responses but also, more importantly, orchestrates the immune system toward the establishment of durable AIM, a paradigm shift essential for overcoming adaptive resistance in cancer immunotherapy.

### Metabolic intervention: the fourth signal empowering AIM

Beyond the classic three-signal activation model, the intrinsic metabolic reprogramming of cells acts as a “fourth signal”, critically determining T-cell fate, particularly the choice between terminal exhaustion and the development of durable AIM [365]. Crucially, the development and maintenance of memory T cells depend on distinct metabolic programs characterized by enhanced oxidative phosphorylation, fatty acid oxidation, and mitochondrial fitness [366]. Metabolic pathways not only provide energy and biosynthetic precursors for T cells but also, importantly, act as important signaling molecules that directly control T-cell differentiation, function, and survival through epigenetic modifications, transcriptional regulation, and signal transduction [367–369]. Given that exhausted T cells exhibit unique metabolic defects (e.g., mitochondrial dysfunction and dysregulated nutrient sensing), metabolic intervention emerges as a powerful strategy to counteract immunoediting by correcting these defects and providing the metabolic foundation for AIM [370, 371]. To this end, several metabolic nodes can be targeted. Parallel to these T-cell-intrinsic strategies, a complementary approach aims to relieve immunosuppressive signals originating from the TME. One such pathway is the adenosine pathway, in which ATP is converted by the ectonucleotidases CD73 and CD39 into adenosine. Adenosine then signals through A2A receptors on T cells, dampening their activation and promoting exhaustion. Accordingly, anti-CD73 antibodies and A2A receptor antagonists have been developed to block this inhibitory axis [372]. Similarly, IDO inhibitors prevent the conversion of tryptophan to kynurenine, a metabolite known to suppress effector T-cell function and promote regulatory T-cell differentiation [373]. Collectively, these metabolic pathway inhibitors function as a form of metabolic checkpoint blockade, alleviating tumor-mediated suppression and synergizing with existing immunotherapies to support durable AIM. Drugs such as metformin activate the energy sensor AMPK, thereby promoting mitochondrial biogenesis and function—a metabolic cornerstone for memory T-cell development that supports the transition from exhaustion to a memory-precursor state [374]. Similarly, targeted regulation of mitochondrial dynamics (e.g., by inhibiting Drp1) can improve metabolic efficiency [375]. Beyond mitochondrial support, the reprogramming of amino acid metabolism breaks metabolic checkpoints to relieve the inhibition of exhausted T cells and potentiate immunotherapy [376]. For instance, providing α-ketoglutarate to inhibit glutaminase promotes epigenetic reprogramming, which is beneficial for T-cell memory formation and functional recovery [377, 378]. L-arginine supplementation enhances mitochondrial metabolism to promote the long-term survival and memory characteristics of T cells [379]. Concurrently, reprogramming one-carbon metabolism (e.g., serine or methionine metabolism) supports epigenetic modifications that promote T-cell proliferation and maintenance of stemness [380, 381]. Furthermore, regulating lipid metabolism to prevent excessive lipid accumulation can increase T-cell persistence and meet the demand for fatty acid oxidation in memory T cells [382–384]. For example, liver X receptor agonists promote cholesterol efflux to enhance TCR signaling [385]. Preclinically, combining such AMPK agonists or mitochondrial enhancers (e.g., spermidine) with ICIs has synergistic effects on restoring T-cell function and inhibiting tumor growth [386–388]. Restoring mannose metabolism enhances T-cell antitumor activity and limits exhaustion by promoting stem-like programs through preserving Tcf7 expression and epigenetic stemness, offering a potential strategy for improving ICT [389]. Despite this promise, the translation of metabolic interventions to robustly support AIM faces specific challenges. Metabolic plasticity in both tumors and T cells can drive adaptive resistance to single-pathway inhibition, necessitating rational combination strategies. Moreover, metabolic modulators often exert divergent effects across T-cell subsets critical for AIM (e.g., effector vs. memory-precursor cells), underscoring the need for subset-selective targeting. Therefore, future efforts must prioritize the development of subset-selective metabolic modulators  and the identification of metabolic biomarkers that predict  which patients can leverage metabolic reprogramming to support  durable AIM, thereby enabling metabolism-targeted personalized immunotherapy.

## Monitoring adaptive resistance immune memory dynamics in cancer immunotherapy

Prior to therapy initiation, the establishment of a comprehensive baseline immune profile of the tumor and its microenvironment forms the crucial foundation for predicting both the likelihood of initial response and the risk of adaptive resistance to cancer immunotherapy. This prognostic evaluation relies on integrating intrinsic tumor characteristics (e.g., TMB and PD-L1 expression) with key microenvironmental features, particularly the density, distribution, and functional status of TILs, with an emphasis on CD8⁺ T cells. For example, a melanoma patient presenting with high TMB, robust tumor PD-L1 expression, and a biopsy showing abundant, functionally active CD8⁺ T cells infiltrating tumor nests exemplifies a classic “immune-hot” profile, which strongly predicts a high potential for a profound initial response to immunotherapies such as ICIs [32, 390]. However, since adaptive resistance evolves dynamically through immunoediting, static baseline biomarkers alone cannot adequately capture its progression or assess the development of durable AIM. Longitudinal, multiparametric monitoring during treatment is essential for tracking key biological transitions in real time. This integrated approach should encompass several dimensions.

First, in a patient with NSCLC who initially responded well, serial liquid biopsies tracking circulating tumor DNA (ctDNA) may reveal a significant decrease after two treatment cycles, which may indicate an early response [391, 392]. A subsequent increase in ctDNA levels, even in the absence of radiographic progression, can indicate emerging resistant clones, offering a critical window for early intervention. Concurrently, dynamic tracking of the peripheral TCR repertoire may reveal the contraction or exhaustion of previously expanded antitumor T-cell clones, accompanied by an increasing frequency of immunosuppressive MDSCs. These shifts collectively reflect immune remodeling under therapeutic pressure. Critically, evolving monitoring strategies should also anticipate resistance mechanisms to guide targeted interventions. A prime example is in NSCLC with comutations in the STK11 and/or KEAP1 tumor suppressor genes. These genotypes are associated with a myeloid-rich, T-cell-depleted immunosuppressive microenvironment resistant to PD-1/PD-L1 blockade [49, 393, 394]. The phase III POSEIDON trial confirmed that adding durvalumab and tremelimumab to chemotherapy, but not durvalumab alone, improved outcomes in this subgroup, supporting initial combination immunotherapy in genomically defined high-risk patients [395].

The mechanism of tumor–immune interactions is extremely complex, and it is difficult to comprehensively reflect the dynamic full picture of a single biomarker. Future breakthroughs will inevitably rely on the systematic integration of multidimensional, dynamically evolving data streams. To this end, more sophisticated models, such as the “cancer immune atlas”, are needed, integrating comprehensive assessments of multiple indicators, including TMB, PD-L1 expression levels, T-cell infiltration density and spatial distribution (as revealed by digital pathology), specific genomic subtypes (e.g., STK11/KEAP1 mutation status), and key serum cytokine concentrations, to generate an integrated immune score for each patient. Furthermore, spatial transcriptomics technology enables us not only to identify the types of immune cells present in the TME but also to visually present their functional spatial distribution. For instance, whether T cells are isolated at the periphery of immune deserts or immune-excluded regions or have penetrated the core of the tumor and are in an activated and attack state remains unclear.

Ultimately, by leveraging artificial intelligence algorithms to integrate these vast amounts of static and dynamic data, patient-specific “digital twin models” of the tumor immune ecosystem can be constructed. One of the key functions of such models is to dynamically simulate the frequency changes, localization migration, and functional state evolution of memory T-cell precursors and their subpopulations. On the basis of such simulations, the timing of clinical interventions will be more precise. For instance, initiating TIGIT inhibitor treatment when T cells show signs of exhaustion, using CSF-1R inhibitors when MDSC activity increases, or promptly adopting dual ICI strategies on the basis of baseline high-risk genomic features. As a result, the cancer immunotherapy model will shift from a “one-size-fits-all approach” to a truly personalized navigation treatment guided by the function of memory T cells.

## Conclusions and perspectives

### Summary of key points

Adaptive resistance to cancer immunotherapy refers to a dynamic, multifactorial process through which tumors evade immune destruction under therapeutic pressure. It manifests in two distinct clinical patterns: on-treatment progression driven by insufficient tumor-killing capacity and postremission relapse resulting from failure to establish or sustain durable antitumor immune memory. As a central challenge across diverse cancers, it critically limits long-term survival for most patients. This process is driven by dynamically evolving immunoediting, in which tumors deploy both intrinsic (e.g., antigen loss) and extrinsic (e.g., T-cell dysfunction, immunosuppressive TME) adaptations. To counter this challenge, a therapeutic paradigm is proposed to evolve from a model focused on tumor clearance to an intelligent strategy that restores immediate effector function while simultaneously guiding the immune system toward establishing long-lasting AIM. Emerging strategies rationally combine ICIs or ICT with agents that remodel the immunosuppressive TME, reprogram tumor metabolism, or promote optimal T-cell responses. The ability of next-generation therapeutic platforms to induce and sustain AIM warrants further in-depth investigation. Promising candidates include bispecific antibodies that costimulate memory pathways, personalized neoantigen vaccines designed to prime high-quality memory, and advanced engineered cellular therapies equipped with memory-like properties (e.g., stem-like or tissue-resident memory-like CAR-T cells or microenvironment-resistant CAR-T cells). The emphasis shifts from achieving immediate tumor eradication to achieving a functional cure, defined by immune-mediated disease control underpinned by the reversion of T-cell exhaustion and durable AIM.

### Challenges and outlook

Cancer immunotherapy has fundamentally altered the clinical management of cancer, yet adaptive resistance continues to limit the durability of treatment responses. Progress in the field will require a shift in scientific focus—from cataloging resistance mechanisms to actively intervening in the immunoediting process to preserve both immediate effector function and sustained AIM. This evolution in approach reveals several interconnected scientific challenges. A primary obstacle is the insufficient mechanistic understanding of the precise molecular and cellular circuits that drive T-cell dysfunction under therapeutic pressure. Although the immunoediting framework describes the transition from immune-mediated elimination to tumor escape, the precise adaptations that disrupt either effector T-cell fitness or memory T-cell differentiation remain inadequately defined. This gap constrains the identification of predictive biomarkers for both resistance trajectories and hinders the rational design of synergistic combination regimens tailored to the predominant defect. Moreover, therapeutic strategies should extend beyond reversing T-cell exhaustion within the TME to actively engage and expand the systemic reservoirs of AIM precursors, such as TdLN-T_TSM_ and Tpex [396–399]. These populations serve as the cellular basis for long-term immunity, highlighting the need for approaches that can quantify, maintain, and amplify these systemic niches rather than merely reactivating TILs. Prolonged antigen exposure intrinsically limits memory reprogramming in exhausted T cells, indicating that therapeutic efficacy depends on recalibrating the kinetics of T-cell stimulation relative to the TME. Furthermore, therapeutic strategies must become as dynamic as the resistance they aim to counter. Although combination regimens represent improvements over monotherapy, static treatment protocols remain vulnerable to evasion because of evolving tumor adaptations. Next-generation strategies should incorporate adaptive therapeutic frameworks guided by real-time biomarker feedback. This will require integrated monitoring platforms that leverage liquid biopsies, spatial multiomics, and immune profiling to dynamically track the balance between effector responses, exhaustion programs, and AIM establishment. Artificial intelligence-powered predictive models of patient-specific tumor-immune ecosystems could subsequently inform optimal intervention timing and sequencing, such as administering a metabolic modulator when mitochondrial dysfunction is detected or introducing a TIGIT inhibitor upon early signs of Tpex contraction. Compounding this challenge, as current preclinical systems often fail to adequately recapitulate the complexity of human immunoediting and T-cell dysfunction, translational models likewise require refinement. Moreover, the clinical development of multiagent immunotherapies is hindered by the complexity of identifying optimal drug combinations, sequences, and administration schedules while mitigating compounded toxicity. Overcoming these barriers will necessitate novel clinical trial frameworks and biomarker-guided patient stratification strategies that distinguish patients whose resistance mechanism is effector exhaustion from those whose risk lies in memory failure.

Future immunotherapy is transitioning from reactive tumor control to proactive, adaptive strategies that sustain AIM, which is enabled by integrating precision stratification, dynamic monitoring, and adaptive intervention into a unified clinical framework. Effective patient stratification should extend beyond conventional biomarkers to incorporate dominant resistance pathways and baseline immune contexture, thereby enabling the selection of rationally designed combination therapies tailored to intercept specific immunoediting trajectories. Concurrently, dynamic monitoring by leveraging liquid biopsies, multi‑omic profiling, and spatial transcriptomics will track real‑time tumor‑immune coevolution, reveal emergent resistance mechanisms, and guide early intervention before clinical progression occurs. These multimodal data streams, integrated via artificial intelligence, can power clinically actionable “digital twins” that simulate individual tumor‑immune ecosystems, optimizing therapeutic sequencing and timing [400]. Ultimately, such adaptive systems support dynamic dosing strategies (such as intermittent or sequential therapy) designed to preserve T-cell fitness and sustain AIM by avoiding continuous immune pressure. The development of vaccines aimed at enhancing effector T-cell function and cell-based therapies designed to establish durable AIM will further expand therapeutic options. The therapeutic objective will thus shift from maximal cytoreduction toward achieving a functional cure and durable, immune‑mediated disease control that ensures long‑term survival despite minimal residual disease. In conclusion, overcoming adaptive resistance necessitates a fundamental reorientation from maximal immune pressure to intelligently orchestration of immunoediting. Therapeutic success should be redefined not by initial tumor regression but by inducing and sustaining a functional AIM state, which requires multidisciplinary integration of the T-cell-mediated antitumor response with dynamic monitoring and adaptive interventions. Prioritizing the durability and quality of the immune response moves us toward lasting functional cures.
